# Synergistic HMGN1 and VP64 Fusions Potentiate High‐Precision and PAM‐Flexible Base Editing

**DOI:** 10.1002/advs.76047

**Published:** 2026-06-11

**Authors:** Xi Luo, Yuyao Qu, Zhengyan Ye, Zheng Li, Yuanyan Zhang, Longjiong Luo, Shaokang Li, Wei Zhao, Ming Wang, Ralph Bock, Jianmin Wan, Junjie Tan

**Affiliations:** ^1^ State Key Laboratory of Crop Genetics & Germplasm Enhancement and Utilization Collaborative Innovation Center for Modern Crop Production co‐sponsored by Province and Ministry Nanjing Agricultural University Nanjing China; ^2^ Zhongshan Biological Breeding Laboratory Nanjing Jiangsu China; ^3^ Department of Plant Pathology Nanjing Agricultural University Nanjing China; ^4^ Department of Organelle Biology, Biotechnology and Molecular Ecophysiology Max‐Planck‐Institut für Molekulare Pflanzenphysiologie Potsdam‐Golm Germany

**Keywords:** base editing, biotechnology, CRISPR, cytidine deaminase, gene therapy, genome editing, HMGN1, precise editing, SpRY, VP64

## Abstract

RNA‐guided CRISPR‐derived base editors (BEs) have revolutionized genome editing by enabling targeted base substitutions. However, their application is frequently constrained by the stringent requirement for PAM sequences and low editing precision (bystander editing). Here, we present a robust strategy to overcome these limitations by coupling SpRY, a near‐PAM‐less Cas9 variant, with truncated CDA1 cytidine deaminases. While this combination enables precise editing of virtually any cytosine in the genome, it initially exhibited suboptimal efficiency. To address this, we systematically screened a diverse panel of candidate DNA‐binding proteins and identified that the synergistic fusion of HMGN1 and VP64 substantially enhances editing activity without compromising precision. Importantly, this enhanced editing efficiency was achieved without markedly increasing off‐target effects. Our new BEs demonstrated robust performance not only in yeast but also in rice, suggesting broad applicability in gene therapy, precision breeding, and fundamental research.

## Graphical abstract

A novel CDA1Δ‐SpRY architecture fused with HMGN1 and VP64 yields a nearly PAM‐less base editing platform. By focusing cytosine conversion predominantly at position −18, this synergistic complex ensures highly precise targeting. Demonstrating enhanced efficiency across diverse models, including yeast and rice, the platform offers a robust solution for comprehensive eukaryotic genome engineering.

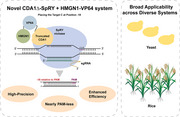

## 1 Introduction

CRISPR‐Cas systems act as a bacterial adaptive immune system to protect their hosts from invasive nucleic acids such as viruses and plasmids [1, 2, 3, 4, 5]. The CRISPR RNA is responsible for recognizing the target site via complementary base pairing, thus determining the site specificity, while the Cas protein, consisting of two nuclease domains, cleaves both strands of the target DNA [2, 3, 6]. The Cas9 enzyme from *Streptococcus pyogenes* (SpCas9) represents a popular tool for genome editing in various organisms, and it has been successfully repurposed for a wide range of applications [2, 4, 7, 8, 9]. When the DNA is cleaved by generation of double‐strand breaks (DSBs), the endogenous cellular DNA repair mechanisms are activated, especially non‐homologous end joining (NHEJ), an efficient but error‐prone DSB repair pathway operating in most cells. As a result, random insertions or deletions (indels) can occur at the target sites, thus generating gene knockouts and loss‐of‐function alleles [6, 10].

Conventional CRISPR‐Cas tools have limited applicability in gene therapy and precision breeding, which typically require the introduction of point mutations rather than loss‐of‐function mutations [11, 12]. For example, more than 50% of human hereditary diseases are caused by point mutations [13]. Therefore, significant efforts have been made toward re‐engineering CRISPR‐Cas systems for the introduction of specific DNA changes [14, 15]. For example, Cas nucleases can be co‐delivered with an exogenous DNA template that contains the desired genomic alterations flanked by sequences homologous to the target site [16]. Once a DSB is generated, the cellular homology‐directed repair (HDR), a high‐fidelity DNA repair pathway, can use the DNA template and recombine it into the DSB site. Although HDR enables a wide range of edits, its editing efficiency is very low in most cell types. Moreover, HDR relies on the availability of donor DNA as a repair template, which can be utilized by other DNA repair pathways such as NHEJ and result in unintended mutations [6, 9, 10, 17]. In recent years, CRISPR base editors (BEs) have been developed by fusing a catalytically impaired Cas nuclease with a deaminase enzyme that changes a nucleobase to an alternative base, thus converting Cas endonucleases into programmable nucleoside deaminases [18, 19, 20, 21]. BEs facilitate the introduction of C‐to‐T, A‐to‐G, and at lower efficiency, also C‐to‐G or A‐to‐Y (Y = C or T) point mutations without producing a DSB in the target DNA or requiring a donor DNA template [18, 20, 22, 23, 24, 25]. BEs are widely applicable tools for precise base substitution in vivo. They enable the correction of disease‐causing point mutations in humans or the introduction of single‐nucleotide changes that underlie quantitative trait loci (QTLs) linked to complex traits in animal or plant breeding [11, 26, 27, 28, 29, 30, 31].

One of the key drawbacks in the applicability of current BEs arises from their relatively wide activity window, also known as low editing precision. For instance, canonical cytosine BEs can potentially edit all Cs located within an approximately 4–17 nucleotide activity window, often resulting in bystander editing [6, 7, 29, 32, 33, 34]. This poses a significant problem when applying BEs to correct disease‐causing mutations in the human genome since most disease‐associated alleles, such as those found in the *HBB* locus for *β*‐thalassemia, the *APOE4* gene linked to Alzheimer's disease, and the *TYR* locus related to oculocutaneous albinism, have multiple bystander Cs within the activity window [18, 35, 36]. Avoiding undesired base editing and the associated amino acid changes remains a major challenge in genome editing. Furthermore, recent studies have suggested that even synonymous substitutions (that do not alter the encoded amino acid) can have strong non‐neutral effects [37]. Thus, bystander editing poses a serious risk, especially in clinical applications of base editing.

Prime editing represents a powerful and versatile genome editing paradigm that couples a reverse transcriptase (RT) fused Cas nickase with a prime editing guide RNA (pegRNA) [38]. This system is distinguished by its ability to install all 12 types of single‐base substitutions and small insertions/deletions without inducing DSBs or relying on donor DNA. Despite these unprecedented capabilities, the editing efficiency of PEs is generally lower than that of BEs, particularly for simple transition mutations (e.g., C·G to T·A conversions) [39]. Furthermore, the implementation of PE is constrained by its complex design requirements involving the optimization of primer binding sites (PBS) and reverse transcriptase template (RTT) sequences. The large molecular size of PEs also poses challenges for viral delivery vectors such as dual‐AAV systems [40, 42], often precluding the inclusion of efficiency‐boosting elements like hMLH1dn [43]. Consequently, while PEs excel in versatility and product purity, CBEs remain the preferred tool for generating high‐efficiency C‐to‐T transitions, provided that their intrinsic limitations regarding targeting scope and editing precision can be effectively addressed.

We have previously established high‐precision CBEs by fusing nCas9 nickase to truncated variants of CDA1, an AID homologue from sea lamprey. Through the characterization of various truncation mutants, we identified specific architectures that predominantly edit the C_−18_ position relative to the PAM [33]. Furthermore, by incorporating Cas9 variants with relaxed PAM recognition, we expanded the targeting scope to include NG sites [34]. However, the continued dependence on G‐containing PAMs still renders many genomic loci inaccessible for precision editing. In this study, we sought to overcome this critical constraint and establish a universally applicable high‐precision base editing platform. To this end, we coupled nSpRY [44], a near‐PAM‐less Cas9 variant, with optimized truncated CDA1 domains to enable unrestricted targeting. Recognizing that PAM‐flexible variants often compromise catalytic activity, we undertook a systematic screening of a diverse panel of DNA‐binding proteins. Crucially, we discovered that synergistic fusions of specific factors (e.g., HMGN1 and VP64) dramatically potentiate editing efficiency without sacrificing the intrinsic precision of the system. We demonstrate the robust efficacy of this strategy in both yeast and rice cells, establishing a versatile toolkit that significantly broadens the scope of base editing for therapeutic development, precision breeding, and functional genomics.

## 2 Results

### 2.1 Expanding the Base Editing Scope of High‐Precision CBEs to Recognize PAM‐Less Sites

**FIGURE 1:**
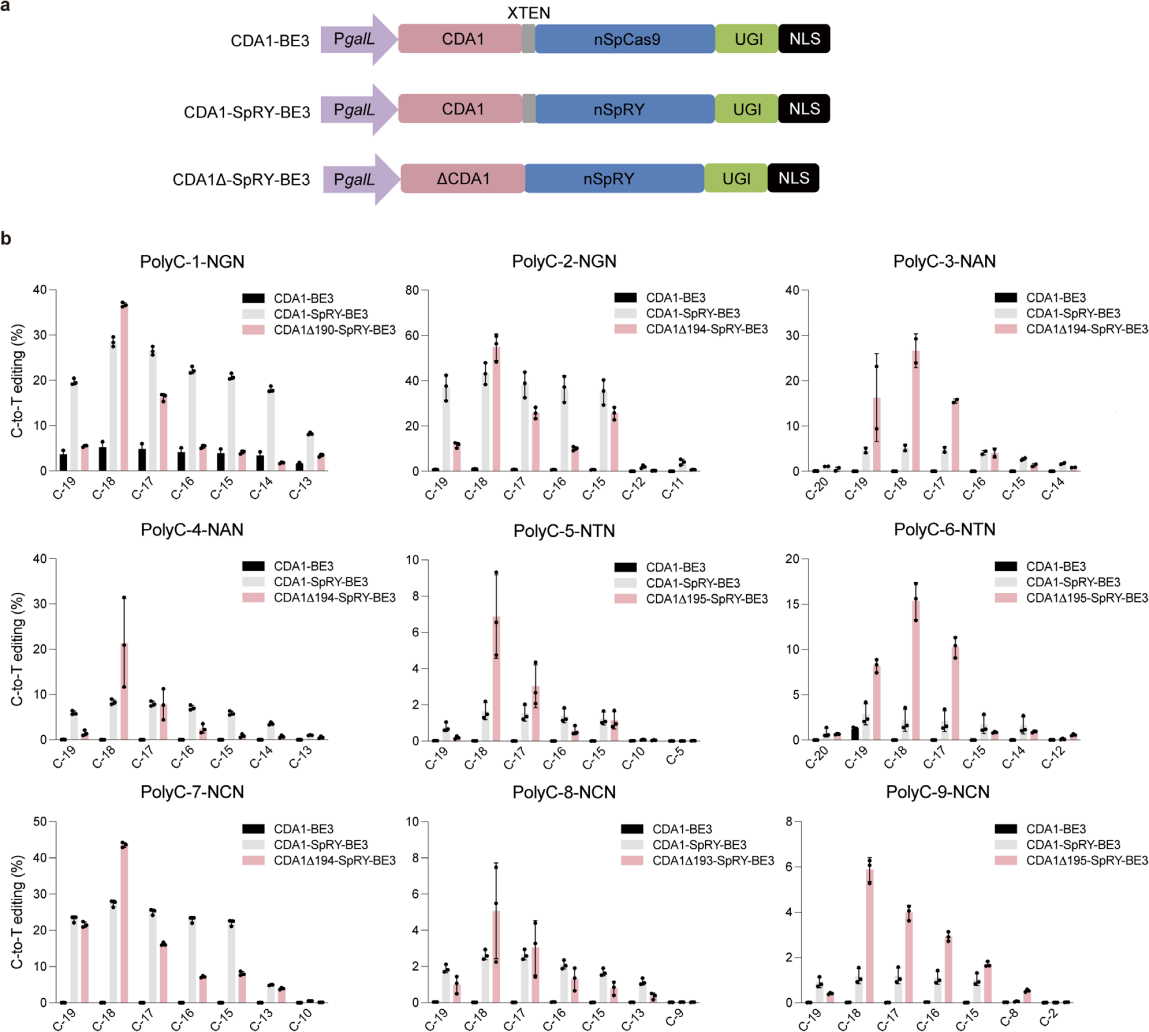
High‐precision C‐to‐T editing in PAM‐less targets. (a) Structure of CDA1‐BE3, CDA1‐SpRY‐BE3 and BEs with CDA1 truncations. nSpCas9: *Streptococcus pyogenes* Cas9 nickase with the mutation D10A; nSpRY: a nCas9 variant with highly flexible PAM specificities [44]; XTEN: synthetic linker sequence [18]; UGI: uracil DNA glycosylase inhibitor; NLS: nuclear localization signal. (b) Base editing outcomes of SpRY BEs at nine target sites lacking an NGG PAM motif (for sequences, see Supplementary Table S1). The BEs harbor selected C‐terminally truncated versions of CDA1 (for additional tested sites and the complete deletion series, see Supplementary Figures S1–S4). The x‐axis shows the Cs in the protospacer sequence with their positions relative to the PAM. The y‐axis represents the percentage of total sequencing reads with the target C converted to T. Values and error bars represent the mean and standard deviation of three independent biological replicates. The source data for (b) are provided as a Source Data file.

In previous work, we have developed CDA1‐based high‐precision CBEs that preferentially edit the position C_−18_ relative to NGG or NGN PAM sequences [33, 34]. However, these BEs still largely rely on the presence of at least one specific nucleotide (G in NGN) in the PAM in proper distance from the target nucleotide. Consequently, precise base editing of nucleotides that do not meet this requirement remains challenging. The engineered highly PAM‐flexible Cas9 variant designated SpRY has been developed [44]. It recognizes NRN and NYN as PAM sequences (with R = A or G and Y = C or T), thus enabling recognition of nearly any NNN sequence as potential PAM [45]. To determine if a near‐PAM‐less Cas9 such as SpRY can be combined with our high‐precision BEs to further broaden their DNA‐targeting range, we replaced nCas9 with nSpRY in our previously described high‐precision BEs (Figure 1a). As a deaminase domain, we used both the full‐length CDA1 and a series of truncated versions lacking 13–20 amino acids from the C‐terminus. Previous research had revealed that this range of C‐terminal deletions, when fused to nCas9, resulted in the highest level of editing precision without compromising the editing activity [33, 34]. In total, seven BEs were produced: the full‐length CDA1 (with an XTEN linker) and six truncated CDA1 variants fused to the N‐terminus of the nSpRY variant (CDA1, CDA1Δ195, CDA1Δ194, CDA1Δ193, CDA1Δ192, CDA1Δ190, and CDA1Δ188‐SpRY‐BE3). The canonical CDA1‐BE3 was used as the control (Figure 1a). We intentionally omitted the XTEN linker in these truncated variants. This linkerless architecture utilizes the inherent flexibility of the CDA1 terminus to minimize the spatial reach of the deaminase domain and achieve a highly restricted editing window focused primarily at the C_−18_ position [33].

For all BEs, we designed 12 non‐NGG target sites that included multiple consecutive cytidines within the activity window to determine their editing profiles (Supplementary Table S1). Oligo(C) motifs, which required maximum discriminatory power to achieve specific editing of a single C, have proven to be the most rigorous way to assess editing precision [29, 46]. Editing efficiency and precision were initially evaluated by dideoxy chain termination sequencing of amplified PCR products (Supplementary Figures S1–S4). The best‐performing BE was subsequently subjected to in‐depth quantitative characterization using high‐throughput next‐generation sequencing (Figure 1b).

The results showed that, while the full‐length CDA1‐SpRY‐BE3 conferred base editing within a wider window ranging from C_−14_ to C_−19_ across all tested target sites, SpRY BEs harboring CDA1 truncations showed 1 to 2 nt activity windows with the maximum editing efficiency observed at position C_−18_, similar to previously described truncated CDA1‐based BEs. At most tested sites (PolyC‐1‐NGN, PolyC‐2‐NGN, PolyC‐4‐NAN, PolyC‐5‐NTN, PolyC‐7‐NCN, and PolyC‐8‐NCN), CDA1Δ‐SpRY‐BEs exhibited very high discriminatory power, with the C_−18_ position being edited two to three times more efficiently than the second most recognized position (C_−17_ or C_−19_).

To systematically define the editing window, we performed a quantitative analysis by extracting and averaging the position‐specific cytosine editing frequencies across 86 evaluated target sites. As visualized in the Supplementary Figure S5, aggregated line plots spanning the protospacer region were generated. By applying a 50% maximum efficiency threshold line consistent with established methodologies [18], we objectively determined the functional editing window for each variant. This comprehensive analysis clearly substantiates the narrowed spatial profile of our optimized truncated constructs compared to the canonical editor.

Based on this quantitative evaluation we identified the specific variant that provides the most favorable architectural balance. The data demonstrates that variants 195 and 194 retain the highest relative editing efficiencies, yielding average increases of 3.11‐fold and 2.85‐fold, respectively. However, a detailed analysis of their normalized editing window profiles reveals a critical distinction in spatial restriction. The variant 195 profile demonstrates a broadened primary editing window reaching the 50% activity threshold at the C_−19_ position, alongside a secondary distal bystander peak at the C_−6_ position. In contrast, the 194 variant eliminates both the distal bystander activity at C_−6_ and the boundary expansion at C_−19_, thereby maintaining a narrowed editing window. By securing consistent on‐target activity while effectively preserving the restricted editing profile, the CDA1Δ194 truncation was consequently defined as our best‐performing construct for subsequent evaluations.

Interestingly, at all tested sites, we observed that SpRY‐BEs with optimized CDA1 truncations displayed much higher editing efficiency at position C_−18_ than the full‐length canonical SpRY BE, especially for NHN PAMs (H = A, T or C), with an up to 6.11‐fold increase in efficiency. A possible explanation for this finding may be linked to decreased SpRY‐based self‐editing, given that high‐precision SpRY‐BEs with a substantially narrower editing window will reduce the probability of introducing multiple mismatches into the 20‐bp spacer upstream of the sgRNA scaffold, which would cause strong decreases in editing efficiency. To experimentally validate this mechanistic hypothesis, we conducted deep sequencing on the sgRNA expression plasmids to quantify self‐editing frequencies (Supplementary Figure S6). These analyses revealed that the full‐length base editor induced broad self‐editing across multiple cytosines spanning positions C_−15_ to C_−19_, whereas our optimized truncated variant restricted self‐editing primarily to the C_−18_ position (Supplementary Figure S6a). To further quantify the impact of this difference on sgRNA integrity, we classified individual sgRNA spacer reads according to the number of C‐to‐T substitutions they contained. This analysis showed that CDA1‐SpRY‐BE3 generated a substantial fraction of sgRNAs carrying multiple mismatches, whereas CDA1Δ194‐SpRY‐BE3 predominantly produced sgRNAs containing only a single mismatch, despite its higher self‐editing activity at C_−18_ (Supplementary Figure S6b–d). Based on well‐established CRISPR targeting principles [2], multiple mismatches within the spacer region significantly impair DNA binding and subsequent editing activity, whereas a single distal mismatch remains well tolerated. Consequently, by limiting the accumulation of multiple mutations within the sgRNA spacer, the narrowed editing window of the truncated variant may help protect the targeting complex from efficiency loss. It is noteworthy that, on average, the SpRY‐BEs exhibited higher editing efficiency at the PAM motif NRN compared to NYN (29.17% vs. 16.56%, Figure 1b), in line with previous reports [44]. To comprehensively evaluate this PAM flexibility, we compiled target editing efficiencies across the NGN, NAN, NCN, and NTN PAM categories into a dedicated comparative analysis (Supplementary Figure S7). The results show that the optimized base editors demonstrate consistent overall activity at NGN and NAN PAM sites, whereas editing frequencies at NCN and NTN PAMs are comparatively lower. This trend accurately reflects the inherent biochemical binding preferences of the SpRY nuclease variant itself.

Three Cas9 variants, Cas9‐NG, SpG and SpRY, have been reported to efficiently tolerate NG PAM sequences. We next compared the ability of these three Cas9 variants to induce precise base editing when combined with optimized CDA1 truncations. To this end, six target sites harboring NG PAMs were designed and the editing profiles of the three BEs were determined. The best‐performing BE for each target site was subsequently quantitatively assessed by high‐throughput sequencing (Supplementary Figure S8). In terms of editing precision, we did not observe pronounced differences between the BEs derived from the three Cas9 variants. When fused with the optimized truncated version of CDA1, all three exhibited a narrow activity window of approximately 1 to 2 nucleotides and achieved maximum editing efficiency at the C_−18_ position. However, SpRY‐based BEs displayed relatively low editing efficiency in three out of six tested sites in comparison to the other two Cas9 variants (Supplementary Figure S8). Thus, Cas9‐NG‐ and SpG‐based BEs are strongly recommended for efficient base editing on NG PAM sites.

Cytidine base editors have been reported to cause significant off‐target effects throughout the genome in an sgRNA‐independent manner [47, 48], raising concerns about their practical application. To investigate off‐target editing, we treated yeast cells with CDA1‐BE3, CDA1‐SpRY‐BE3, CDA1Δ195‐SpRY‐BE3, CDA1Δ194‐SpRY‐BE3, CDA1Δ190‐SpRY‐BE3, and a no BE control, using an sgRNA targeting the *Can1* site (Supplementary Table S1). Canavanine selection was used to isolate colonies with on‐target editing events, and whole‐genome sequencing was performed on mixed cultures from three different transformed colonies (see Methods; primers are listed in Supplementary Table S2). As expected, the four SpRY BE variants showed a comparable number of indels to the canonical CDA1‐BE3 and the no BE control (Supplementary Figure S9a). By contrast, upon analyzing single‐nucleotide variants (SNVs), we observed that full‐length CDA1 fusions induced significantly more SNVs than the control, consistent with previous reports on the genome‐wide off‐target effects of CDA1‐based BEs [34], whereas the three SpRY BEs with truncated CDA1 versions displayed fewer SNVs than full‐length CDA1 fusions, although slightly more than the control (Supplementary Figure S9b). We also examined the mutation types and found that C‐to‐T (G‐to‐A) transitions were more frequent in CDA1‐BE3 and CDA1‐SpRY‐BEs compared to the control and the three truncated BEs. The largest truncation, CDA1Δ190‐SpRY‐BE3, displayed a particularly low transition level that was almost comparable to that of the negative control (Supplementary Figure S9c). These findings demonstrate that high‐precision BEs can minimize non‐specific editing at off‐target sites.

Taken together, our results suggest that, by combining the Cas9 variant SpRY with CDA1 truncations, high‐precision base editors can be obtained that significantly broaden the scope of base editing by facilitating editing in near‐PAM‐less sequence contexts.

### 2.2 Precise Base Editing of Any User‐Chosen Single Cytidine in the Genome

**FIGURE 2:**
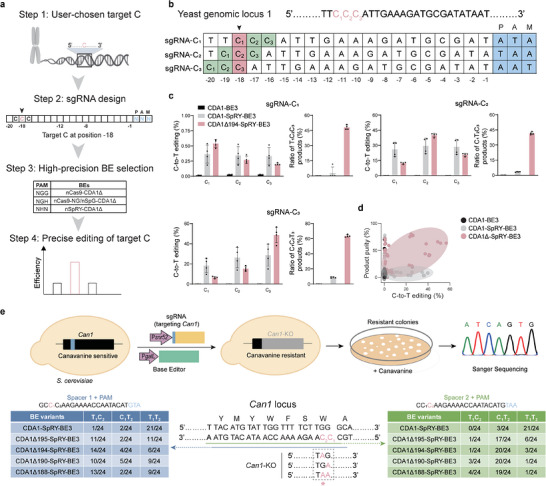
Precise base editing for any user‐chosen single cytidine in genomic DNA by BEs with CDA1 truncations. (a) Workflow for precise base editing of a chosen cytidine in genomic DNA by BEs with CDA1 truncations. The workflow is illustrated as a straightforward four‐step process. (b) A target site with three consecutive cytidines in the yeast genome was selected to demonstrate the process. The cytidines were labeled as C_1_, C_2_, and C_3_ from 5' to 3', and each cytidine was individually targeted (highlighted in red), while the other two served as bystander bases (highlighted in green). This setup facilitated investigation of the ability of CDA1‐truncated BEs to distinguish between multiple cytidines. To perform precise base editing at each of the three target cytidines, the corresponding sgRNA was designed, placing the target C at position −18. Three distinct sgRNAs were designed, named sgRNA‐C_1_, sgRNA‐C_2_, and sgRNA‐C_3_, and their corresponding PAM sequences are highlighted in blue. (c) Base editing outcomes for the three cytidines were evaluated by next‐generation sequencing, analyzing the fraction of desired editing products for each sgRNA. Values and error bars represent the mean and standard deviation of four independent biological replicates. (d) Scatter plot correlating the editing efficiency at position C_−18_ with the corresponding product purity for each of the five tested target sites. (e) Base editing precision was further assessed in individual yeast colonies selected for canavanine resistance. L‐canavanine is a highly toxic analog of the proteinogenic amino acid arginine, and mutations inactivating the uptake protein Can1 confer resistance to canavanine. A DNA fragment from the *Can1* coding sequence was selected as the target site, where two cytidines (marked as C_1_ and C_2_ in red) can cause Can1 inactivation (*Can1‐KO*) if either of them or both undergo C‐to‐T mutation, resulting in resistance to canavanine. To ensure precise editing of the two cytidines, two individual 20‐bp spacers, spacer 1 (blue) and spacer 2 (green), were derived from the downstream sequence. Yeast cells were transformed with plasmids expressing the base editors and their corresponding sgRNAs. For each transformation, 24 canavanine‐resistant colonies were randomly picked followed by sequencing of the *Can1* locus. The first row of the table lists the three major edited product types. The numbers of colonies for each product type are provided for all BEs tested. Source data for (c) and (d) are provided in the Source Data file.

Given the ability of SpRY‐BEs with CDA1 truncations to selectively edit positions at C_−18_ relative to any (NNN) PAM sequence, it should be feasible to precisely edit any cytidine in the genome using CDA1‐based BEs by following a straightforward four‐step design (Figure 2a): (i) acquire the genomic sequence and identify the target cytidine, (ii) position the target C at −18 bp (C_−18_) to define the 20‐bp spacer and identify the PAM motif, and design an sgRNA for the target site, (iii) select the appropriate high‐precision BE based on the PAM sequence context, considering that nCas9 BEs with CDA1 truncations (nCas9‐CDA1Δ) are optimal for NGG PAMs, Cas9‐NG or SpG BEs with CDA1 truncations (nCas9‐NG/nSpG‐CDA1Δ) are preferred for NGH PAMs, whereas for PAMs that do not fall into either of these two classes, SpRY BEs with CDA1 truncations (nSpRY‐CDA1Δ) are the best option, and (iv) perform precise base editing by selecting the optimal truncated version of CDA1.

We then tested the robustness of this approach by randomly selecting a target site in the yeast genome that contained three consecutive cytidines (labeled C_1_, C_2_, and C_3_ in 5' to 3' sequence direction). Each of the three cytidines was selected as target C for base editing, with the other two Cs acting as bystanders. Based on the above criteria, three sgRNAs were designed: sgRNA‐C_1_, sgRNA‐C_2_, and sgRNA‐C_3_, each targeting a specific cytidine (C_1_, C_2_, or C_3_), with the target C located at position −18 in the 20‐bp spacer, upstream of the resulting PAM sequence (Figure 2b). Next, based on the identified PAM sequence, the appropriate BEs were selected. Since all three target sites contained NHN PAM sequences (ATA, TAA, and AAT for target C_1_, C_2_, and C_3_, respectively), SpRY‐BEs with CDA1 truncations were chosen. The best‐performing CDA1 truncation, CDA1Δ194, was selected to construct the high‐precision BE, CDA1Δ194‐SpRY‐BE3, while two canonical CDA1‐based BEs, CDA1‐BE3 and CDA1‐SpRY‐BE3, were used as controls. The results obtained demonstrated that, compared to the controls, which displayed lower editing efficiency and precision, CDA1Δ194‐SpRY‐BE3 achieved higher editing efficiency and precision, with the maximum editing efficiency at each target C being 1.05 to 7.41‐fold higher than that of the bystander Cs. Importantly, when analyzing the ratio of products with only the target C edited to the total edited products, we observed that high‐precision BEs with CDA1 truncations produced a high proportion of single‐target C‐modified products (41.60%–63.65% of all edited products). By contrast, the controls primarily yielded double or triple‐edited products, and less than 8.7% of the products represented the desired single‐target edits (Figure 2c). To further validate the effectiveness of our approach, we extended our analysis to include four additional randomly selected target sites, and consistent precision editing outcomes were observed across all sites (Supplementary Figures S10–S13). To systematically evaluate both editing efficiency and product purity, we generated a scatter plot correlating the editing efficiency at the C_−18_ position with the corresponding product purity for each individual site (Figure 2d). The data clearly demonstrate that CDA1Δ‐SpRY‐BE3 not only enhances editing efficiency, but also significantly increases product purity, thus further underscoring its potential as a highly precise base editing tool.

Finally, we also determined the base editing outcomes in individual yeast colonies obtained by the canavanine selection method described previously (Figure 2e) [33]. To this end, we selected a target sequence from the *Can1* coding sequence where two cytidines (designated C_1_ and C_2_ in red in Figure 2e) would cause *Can1* inactivation, if either of them (or both) undergo C‐to‐T mutation, thus resulting in resistance to canavanine. We designed two spacers (spacer 1 and 2) for two corresponding sgRNAs to target each of the two Cs. Using SpRY BEs with truncated CDA1 domains, we obtained 11 to 14 colonies (for spacer 1) and 17 to 20 colonies (for spacer 2) out of 24 randomly selected colonies that were homozygous for the allele only edited at the single targeted C. By contrast, the full‐length CDA1‐SpRY‐BE3 yielded only 1 and 3 colonies, respectively, that showed specific editing of C_1_ or C_2_ in a homozygous fashion (Figure 2e).

### 2.3 Improvement of Base Editing Efficiency for High‐Precision SpRY BEs

**FIGURE 3:**
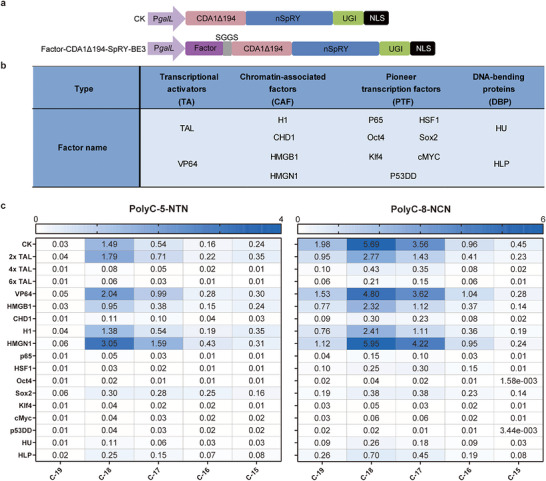
Evaluation of fusions to DNA‐binding proteins for improved base editing efficiencies. (a) Schematic representation of the constructs used to test the effect of different DNA‐interacting factors fused to the N‐terminus of the best‐performing truncated version, CDA1Δ194‐SpRY‐BE3 (CK). (b) Classification of DNA‐interaction factors tested in this study. The factors are grouped into four categories based on their functional roles: Transcription Activators (TA), Chromatin‐associated Factors (CAF), Pioneer Transcription Factors (PTF), and DNA‐Bending Proteins (DBP). (c) Heatmaps showing C‐to‐T editing frequencies at various cytosine positions within two target sites: PolyC‐5‐NTN and PolyC‐8‐NCN. The editing efficiencies were measured for constructs containing the different DNA‐interacting factors, as indicated on the y‐axis. Each column represents a cytosine position (C_−19_ to C_−15_), and editing efficiencies are visualized in shades of blue. Higher frequencies are represented by deeper blue tones, highlighting significant enhancement obtained with specific factors such as HMGN1 and VP64. Values represent the mean of three independent biological replicates. Source data are provided as a Source Data file.

**FIGURE 4:**
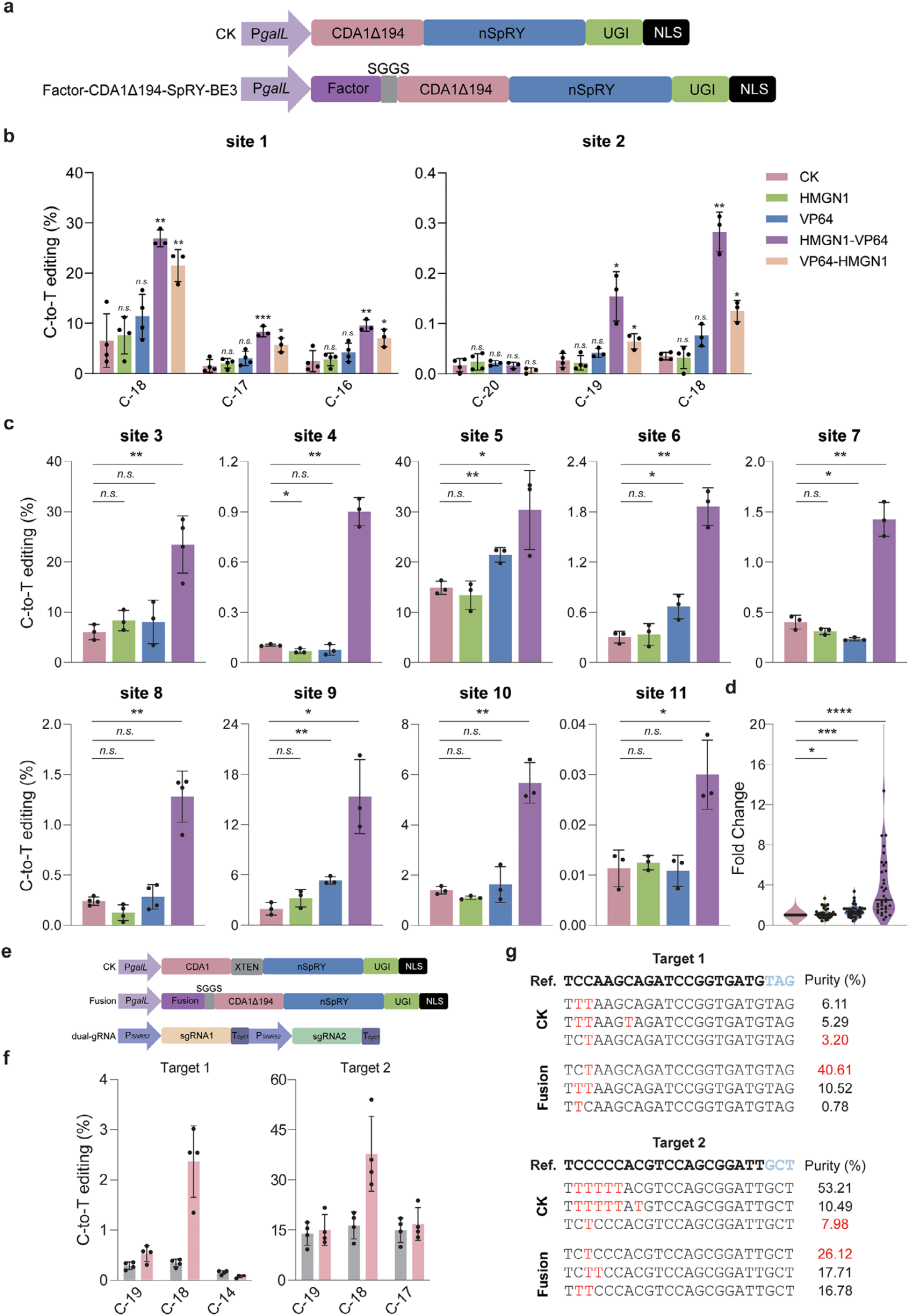
Evaluation of base editing efficiency with different factor fusions. (a) Schematic maps of constructs used to test the effect of different DNA‐interacting protein factors (including HMGN1 and VP64, and combinations thereof) fused to the N‐terminus of CDA1Δ194‐SpRY‐BE3 (CK). (b) Editing efficiencies at two target sites (sites 1 and 2) lacking an NGG PAM motif obtained with CDA1Δ194‐SpRY‐BE3 and different fusions to DNA‐binding proteins (CK: control, HMGN1, VP64, HMGN1‐VP64 fusion, VP64‐HMGN1 fusion). The x‐axis represents the positions of Cs in the protospacer sequence relative to the PAM, and the y‐axis shows the percentage of total sequencing reads where the target C is converted to T. (c) Editing efficiency at position C_−18_ for nine additional target sites (sites 3–11) tested with constructs expressing CK (control), HMGN1, VP64, or the HMGN1‐VP64 fusion. Editing efficiency data for additional sites and all cytosines across these sites can be found in Supplementary Figure S14. (d) Fold change in editing efficiency across all 36 tested sites. Editing efficiency in the CK control was set to 1, and the efficiencies of all other constructs are shown as fold increases relative to CK. The horizontal line in each dataset represents the median fold change. The violin plots illustrate the distribution of editing efficiencies, with the HMGN1‐VP64 fusion showing a strong increase in editing efficiency across the tested sites. (e) Schematic representation of the CDA1‐SpRY‐BE3 (CK) construct, the HMGN1‐VP64 chimeric protein fused to the N‐terminus of the best‐performing truncated BE version, CDA1Δ194‐SpRY‐BE3, and the dual‐gRNA system. (f) Base editing outcomes of SpRY BEs at two target sites (target 1 and target 2; for sequences, see Supplementary Table S1). The x‐axis shows the Cs in the protospacer sequence with their positions relative to the PAM. The y‐axis represents the percentage of total sequencing reads with the target C converted to T. (g) Analysis of the purity of the main editing products for target 1 and target 2. The purities of singly modified C_−18_ products are highlighted in red. Purity (%) is defined as the percentage of sequencing reads containing the edited product among the total edited reads. In (b), (c), and (f), values and error bars represent the mean and standard deviation of three independent biological replicates. Statistical significance in (b)—(d) is indicated as *p < 0.05, **p < 0.01, ***p < 0.001, ****p < 0.0001, *n.s*., not significant (Student's t‐test). The target sites in (b), (c), and (f) were randomly selected across the genome to ensure an unbiased assessment, with the majority located in intergenic regions. Source data for (b), (c), (d), and (f) are provided in the Source Data file.

While the target flexibility of the SpRY variant can significantly broaden base editing capabilities of current high‐precision BEs, their editing efficiency is relatively low, particularly at targets with NYN PAM motifs. Thus, further improvements are needed before these BEs can be widely applied. Previous studies have shown that enhancing the interaction between editing deaminases and DNA can further improve editing efficiency [49]. To explore the potential of enhancing editing efficiency of SpRY‐based editors, we undertook a systematic screening of 15 DNA‐interacting proteins that represent a diverse range of DNA‐binding mechanisms. The set included two potent transcription activators (Transcription Activator‐Like Effector (TAL) and Viral Protein 64 (VP64)), four chromatin‐associated factors (High Mobility Group Box 1 (HMGB1), Chromodomain‐Helicase‐DNA‐Binding Protein 1 (CHD1), Histone H1 (H1), and High Mobility Group Nucleosome Binding Domain Containing Protein 1 (HMGN1)), seven pioneer transcription factors (nuclear factor‐κB *trans*‐activating subunit p65 (p65), Heat Shock Factor 1 (HSF1), Octamer‐binding Transcription Factor 4 (Oct4), SRY‐Box Transcription Factor 2 (Sox2), Krüppel‐like Factor 4 (Klf4), c‐Myc (cMyc), and p53‐Double Domain (p53DD), and two bacterial DNA‐bending peptides (the histone‐like proteins HU and HLP), with the goal to identify factors that are capable of enhancing the performance of CDA1Δ‐SpRY‐BE3 (Figure 3). We individually fused each of these 15 DNA‐interaction factors to the N‐terminus of the best‐performing SpRY‐based cytosine BE version, CDA1Δ194‐SpRY‐BE3, thus generating a series of factor‐CDA1Δ194‐SpRY‐BE3 constructs (Figure 3a). We fused the DNA‐binding domains to the N‐terminus, rather than the C‐terminus, of the base editors, based on our previous finding that N‐terminal fusions do not compromise the editing precision of BEs [33]. Next, we performed efficiency tests on two target sites with an NYN PAM motif. The results revealed that the fusion of most proteins to CDA1Δ194‐SpRY‐BE3 led to a reduction in editing efficiency. However, two factors, HMGN1 and VP64, showed notable improvements. HMGN1 fusion enhanced editing efficiency at both target sites, while VP64 fusion improved editing efficiency at one site and the other site showed similar efficiency to the control (Figure 3c). Next, we tested combinations of VP64 and HMGN1 fusions by generating VP64‐HMGN1‐CDA1Δ194‐SpRY‐BE3 and HMGN1‐VP64‐CDA1Δ194‐SpRY‐BE3 constructs (Figure 4a). Efficiency tests on two endogenous target sites showed a significant boost in editing efficiency when both factors were fused to the BE (Figure 4b). Notably, the HMGN1‐VP64 fusion resulted in a more substantial increase in editing efficiency compared to the VP64‐HMGN1 combination.

To further assess the effectiveness of the fused DNA‐binding proteins in enhancing editing efficiency, we conducted additional tests on 16 endogenous target sites in yeast. All editors maintained high precision, with the highest editing efficiency observed predominantly at C_−18_ (Supplementary Figure S14). In terms of editing efficiency, compared to the control CDA1Δ194‐SpRY‐BE3, single‐factor fusions led to modest improvements at only a few sites. By contrast, the HMGN1‐VP64 combination significantly boosted editing efficiency across all tested sites (Supplementary Figure S14; Figure 4c).

We then expanded the analysis to 36 target sites and determined the fold changes. The single‐factor fusions resulted in average increases of 1.17‐fold and 1.42‐fold for HMGN1 and VP64, respectively, compared to the control. By contrast, the dual‐factor fusions led to a much larger average increase of 4.24‐fold (Figure 4d). These results suggest that fusing functional DNA‐binding motifs or optimizing their conformation can significantly enhance the efficiency of high‐precision base editors.

Next, we evaluated the performance of base editors incorporating the fusion protein in multiplex editing. To this end, two sgRNAs (sgRNA1 and sgRNA2) were co‐expressed from a single dual‐sgRNA vector. The two sgRNAs target different endogenous loci in the yeast genome (target 1 and target 2; Figure 4e). Our results show that base editors containing the fusion protein and harboring the CDA1 truncation confer enhanced editing efficiency, leading to a 6.86‐fold increase and a 2.31‐fold increase in editing at position C_−18_ in target 1 and target 2, respectively, compared to the control editor (Figure 4f). Importantly, these base editors also maintained high precision at both loci, with the most highly edited position being edited 4.41‐fold and 2.51‐fold more efficiently than the second most recognized position for target 1 and target 2, respectively. Further analysis of the edited products revealed that the fusion protein‐harboring base editors selectively edited C_−18_ with high specificity, generating 40.61% C_−18_‐only‐edited products at target 1 and 26.12% at target 2, while the control editor produced only 3.2% and 7.98% C_−18_‐only‐edited products, respectively (Figure 4g). These findings demonstrate the capacity of fusion protein‐containing base editors to enable precise genetic modifications at multiple loci.

To further investigate the generalizability of the synergistic fusion strategy across different base editor architectures, we fused the HMGN1 and VP64 modules to the N‐terminus of an rAPOBEC1‐mediated SpRY base editor. Subsequent evaluation across eleven endogenous target loci in yeast revealed an average 2.47‐fold increase in editing efficiency (Supplementary Figure S15). Although this enhancement is slightly lower than the 4.24‐fold average increase observed with the CDA1 system, these results confirm that the dual‐factor fusion strategy can be effectively extended to other widely used cytidine deaminases to rescue compromised editing activity.

### 2.4 Analysis of Genome‐Wide Off‐Target Editing

Given that HMGN1 and VP64 likely enhance the interaction between BEs and DNA, we next investigated whether they affect off‐target editing. We evaluated off‐target editing in both the single‐factor fusion BEs, HMGN1‐CDA1Δ194‐SpRY‐BE3 and VP64‐CDA1Δ194‐SpRY‐BE3, and the dual‐factor fusions, VP64‐HMGN1‐CDA1Δ194‐SpRY‐BE3 and HMGN1‐VP64‐CDA1Δ194‐SpRY‐BE3. For comparison, we also included the canonical CDA1‐BE3, the near‐PAM‐less BEs CDA1‐SpRY‐BE3 and CDA1Δ194‐SpRY‐BE3, and a no BE control, in combination with an sgRNA targeting the *Can1* locus (Supplementary Table S1). Canavanine selection was used to isolate colonies with on‐target editing events, and whole‐genome sequencing was performed on pooled cultures from three independent transformed colonies (see Methods; primers are listed in Supplementary Table S2).

**FIGURE 5:**
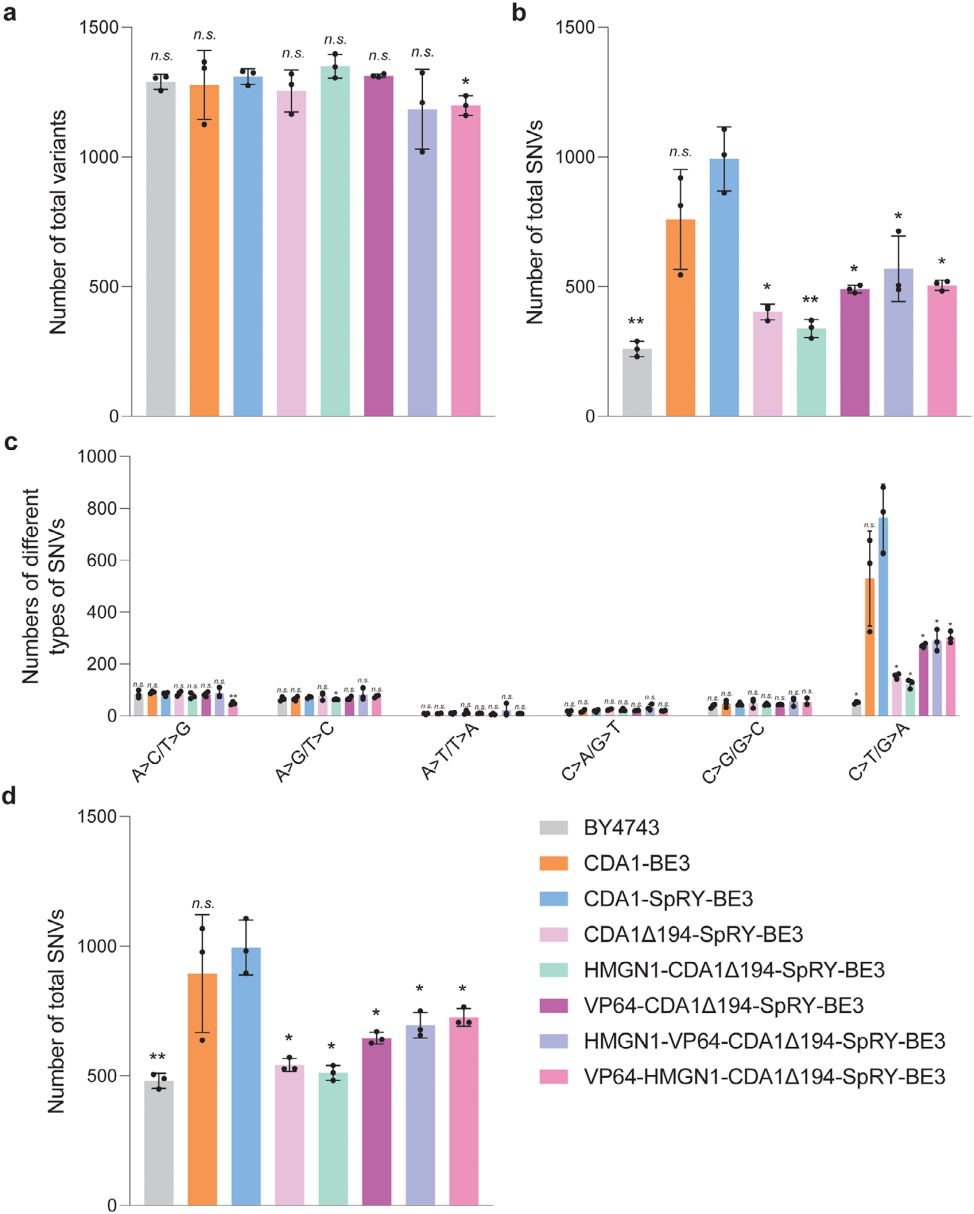
Analysis of off‐target editing. (a–c) Whole‐genome sequencing was performed to assess genetic changes in strains expressing various base editors (BEs), including CDA1‐BE3, CDA1‐SpRY‐BE3, CDA1Δ194‐SpRY‐BE3, HMGN1‐CDA1Δ194‐SpRY‐BE3, VP64‐CDA1Δ194‐SpRY‐BE3, HMGN1‐VP64‐CDA1Δ194‐SpRY‐BE3, and VP64‐HMGN1‐CDA1Δ194‐SpRY‐BE3, as well as a control plasmid lacking a BE construct. (a,b) Comparison of the total number of detected indels (a) and SNVs (b) for the different BE constructs and the control. c Mutation frequencies for different types of SNVs in cells treated with the seven base editors and the control construct. The sgRNA was designed to target the *Can1* locus (Supplementary Table S1). (d) Transcriptome‐wide analysis of off‐target RNA editing. Comparison of the total number of RNA edits in cellular transcripts from cells treated with the seven BEs and the control construct. Values and error bars for (a–d) represent the mean and the standard deviation of three biological replicates. Statistical significance is indicated as *p < 0.05, **p < 0.01, *n.s*., not significant; CDA1‐SpRY‐BE3 was used as the control (Student's *t*‐test). Source data for (a–d) are provided as a Source Data file.

While all SpRY BE variants exhibited comparable numbers of indels to both the canonical CDA1‐BE3 and the no BE control (Figure 5a), analysis of SNVs revealed that all SpRY BEs containing truncated CDA1 versions produced fewer SNVs than the full‐length CDA1 fusions. Notably, the inclusion of VP64 in the fusion led to a slight increase in the number of SNVs. This marginal elevation is likely driven by the VP64 domain altering local transcriptional activity to expose single‐stranded DNA [50]. Nevertheless, the overall SNV frequencies in these fusion constructs remained significantly lower when compared to the untruncated canonical CDA1‐SpRY‐BE3 editor (Figure 5b). When examining the mutation types, we observed that C‐to‐T (G‐to‐A) transitions were more frequent in CDA1‐BE3 and CDA1‐SpRY‐BEs than in the control and the truncated BEs (Figure 5c). To further evaluate sgRNA‐dependent off‐target editing, we utilized Cas‐OFFinder [51] (http://www.rgenome.net/cas‐offinder/) to predict potential off‐target sites for four independent target loci. Targeted next‐generation sequencing of these predicted sites demonstrated that the newly developed base editors maintained off‐target editing frequencies comparable to the control group (Supplementary Figure S16). These results confirm that the fusion strategy does not induce additional sgRNA‐dependent DNA mutations and preserves the targeting specificity.

We also assessed off‐target RNA editing effects by analyzing both total SNVs and specific mutation types including C‐to‐U and A‐to‐I transitions (Figure 5d; Supplementary Figure S17). Compared to the negative control, BEs with full‐length CDA1 exhibited higher off‐target RNA editing primarily driven by C‐to‐U conversions. By contrast, BEs containing CDA1 truncations displayed RNA editing levels comparable to the control, with CDA1Δ194‐SpRY‐BE3 and HMGN1‐CDA1Δ194‐SpRY‐BE3 showing no significant increase, and VP64‐containing fusions displaying only a slight increase in C‐to‐U variants. These results indicate that the fusion of the two DNA‐binding proteins does not significantly enhance off‐target editing, thus preserving the high specificity of the base editors.

### 2.5 Enhanced Base Editing With Truncated CDA1 Variants and DNA‐Binding Protein Fusions in Plant Cells

**FIGURE 6:**
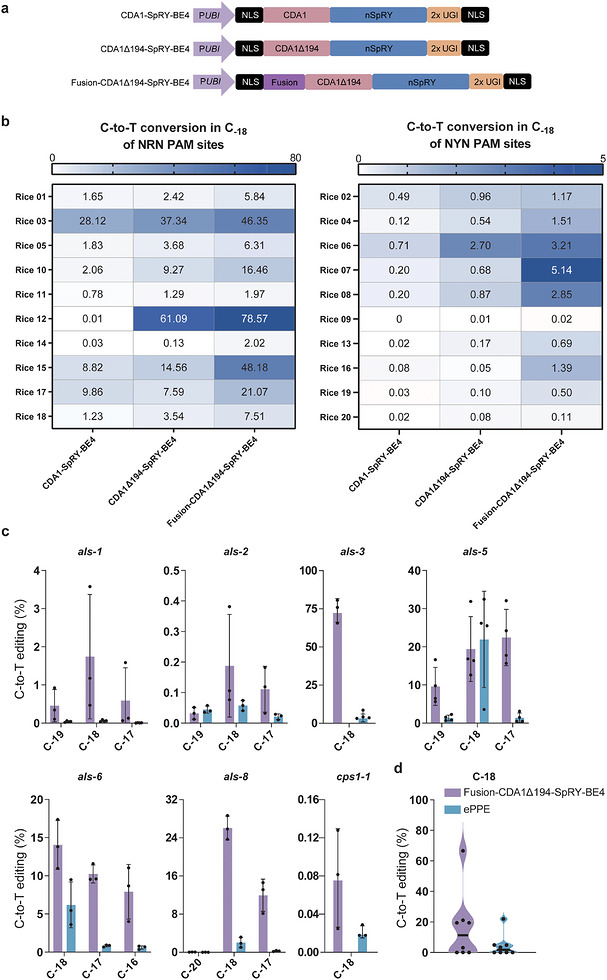
Evaluation of base editing efficiency in plant cells. (a) Schematic representation of the BE constructs CDA1‐SpRY‐BE4, CDA1Δ194‐SpRY‐BE4, and Fusion‐CDA1Δ194‐SpRY‐BE4 tested in rice cells. Each BE was driven by the maize *Ubiquitin* promoter (p*UBI*). See Methods for details. (b) Heatmaps displaying C‐to‐T editing frequencies at position C_−18_ for the three BEs across twenty endogenous target sites in the rice genome (Rice 01–20), including ten NRN and ten NYN target sites. Note that different color scales are used for NRN and NYN sites to visualize the lower efficiency at NYN targets. Values represent the means of three independent biological replicates. (c) Bar graph comparing C‐to‐T editing frequencies between Fusion‐CDA1Δ194‐SpRY‐BE4 and ePPE at seven endogenous target sites in rice cells. Values and error bars represent the mean and the standard deviation of at least three biological replicates. (d) Violin plot summarizing the distribution of C‐to‐T editing efficiencies for Fusion‐CDA1Δ194‐SpRY‐BE4 and ePPE across the seven target sites. The horizontal bar indicates the median efficiency for each base editor. Each dot represents the editing frequency in an individual biological replicate. Source data for (b‐d) are available in the Source Data file.

To further explore the potential of truncated CDA1 SpRY base editors (BEs) and their dual‐factor fusions in enhancing base editing efficiency in cells of higher eukaryotes, we evaluated the editing performance of the best‐performing truncated version, CDA1Δ194‐SpRY‐BE4, and its N‐terminal fusion with HMGN1 and VP64 (referred to as CDA1Δ194‐SpRY‐BE4 and Fusion‐CDA1Δ194‐SpRY‐BE4, respectively), in rice cells. These two BEs were compared to the corresponding full‐length CDA1‐SpRY‐BE4 as control (CDA1‐SpRY‐BE4; Figure 6a). Base editing efficiency was assessed at 20 endogenous target sites in the rice genome (Figure 6b). The results demonstrated that BEs with truncated CDA1 improved editing efficiency at 18 of the 20 sites, with increases ranging from 1.32‐fold to 4617.95‐fold, and a maximum efficiency of 61.09%. Furthermore, fusion with the DNA‐binding proteins led to even greater improvements. Compared to full‐length CDA1 base editors, the fusion constructs showed 1.64‐fold to 5938.93‐fold higher efficiency, with a maximum of 78.57%. When compared to the CDA1Δ194‐SpRY‐BE4, the fusion protein resulted in a 1.18‐fold to 26.87‐fold increase in efficiency across all tested sites. These findings underscore the potential of using truncated CDA1 variants and fusion proteins to enhance base editing efficiency, extending this strategy from yeast to cells of higher eukaryotes. Notably, while editing efficiency was enhanced at all target sites, the efficiency at NYN target sites was substantially lower than at NRN target sites. This highlights the promise of fusion proteins in boosting editing efficiency, although further optimization will be needed to enhance performance at NYN sites.

To further validate the superior performance of our optimized base editors, we conducted a comprehensive comparison with ePPE, a plant‐optimized prime editor [52], across seven endogenous non‐NGG target sites in rice cells (Figure 6c). For ePPE, the pegRNAs were designed by using the online tool (http://www.plantgenomeediting.net/) to convert C_−18_ to T. To ensure a meaningful and objective comparison, both systems were assessed at their respective optimal configurations. The target cytosines were directly positioned at C_−18_, representing the optimal editing window for the base editors. Detailed positional mapping for all evaluated targets relative to both editing mechanisms was provided in Supplementary Table S3. The side‐by‐side comparison revealed that, despite slightly higher bystander editing, our optimized base editor, Fusion‐CDA1Δ194‐SpRY‐BE4, outperformed ePPE in terms of editing efficiency at six of seven tested sites, with editing efficiencies being 2.27‐ to 28.61‐fold higher. Fusion‐CDA1Δ194‐SpRY‐BE4 achieved an average editing efficiency of 18.53%, compared to only 5.31% with ePPE (Figure 6d). In summary, this comparison confirms the enhanced editing capability of our optimized base editors and establishes their potential as superior tools for precision genome editing in plant systems.

## 3 Conclusion

In this study, we have (i) substantially expanded the applicability of our previously developed high‐precision base editors (BEs) to nearly PAM‐less targets, (ii) established a streamlined four‐step approach for PAM‐independent targeting and validated its capability to precisely edit virtually any cytosine, as demonstrated at five randomly selected genomic sites, (iii) identified specific DNA‐binding domains, and particularly their optimal combinations, that strongly enhance editing efficiency without compromising precision, and (iv) demonstrated the robust efficacy of this strategy in both yeast and rice, establishing a versatile toolkit for broad biotechnological applications.

The Cas9 variant SpRY has demonstrated significant potential for expanding the scope of gene editing by greatly relaxing the PAM sequence requirements, thus allowing for editing at a wider range of targets, even including sites that are nearly PAM‐less [44]. Despite these potential benefits, SpRY has been found to have the drawback of targeting its own sgRNA, leading to editing of the spacer region, a phenomenon known as self‐editing [53, 54]. This self‐editing can decrease the efficiency of the intended editing and increase the risk of off‐target effects, as the altered spacer can target new regions in the genome. In this study, we discovered that the combinations of nSpRY and truncated CDA1 domains displayed increased editing efficiency compared to canonical full‐length CDA1‐based SpRY BEs. The occurrence of this editing‐stimulating effect was surprising in that it had not been observed previously in fusions of CDA1 truncations with nCas9 or its variants [33, 34]. We hypothesize that this benefit stems from the drastically narrowed editing window of the truncated deaminase. Structurally, the full‐length CDA1 features a highly flexible C‐terminal extension that acts as a long tether allowing its catalytic domain to access a broad range of cytosines, which inherently compromises precision [33]. Our truncation strategy directly removes this flexible tail to physically shorten the connection to the Cas9 nickase. This compact architecture severely restricts the conformational freedom of the deaminase. By limiting the spatial reach of the catalytic pocket, the enzyme is physically constrained to focus its editing activity. By restricting activity to the C_−18_ position, the likelihood of introducing mutations within the 20‐bp spacer sequence of the sgRNA expression cassette is significantly reduced. By restricting deamination primarily to the C_−18_ position, the truncated editor reduces combinatorial editing within the sgRNA spacer and markedly lowers the proportion of sgRNAs carrying multiple mismatches. This may help preserve a larger pool of functional sgRNAs and partially explain the improved on‐target editing efficiency observed with CDA1Δ194‐SpRY‐BE3. Our findings offer a new strategy to alleviate SpRY‐based self‐editing, which can be extended to other high‐precision BEs (e.g., YE1/YE2/YEE‐BE3 [55], eA3A/A3AΔ‐BE3 [32, 34]) to improve their effectiveness.

The four‐step protocol we propose in this study for precise editing of any cytidine in the genome is mainly based on CDA1‐derived BEs, and combines findings from this and other recent studies [33, 34]. In fact, many engineered BEs have demonstrated superior performance in discriminating between multiple cytidines, especially on target sites with NGG PAMs (e.g., the APOBEC1‐based YE1/YE2/R33A‐BE3 [55, 56] and the APOBEC3A‐based eA3A/A3AΔ‐BE3 [32, 34]). In addition, compared to the wild‐type Cas9 and its derived BEs, Cas9 variants and their corresponding BEs typically display reduced editing efficiency to varying degrees, thus offsetting their advantage of providing increased compatibility with alternative PAM sequences [44, 57]. We, therefore, strongly suggest using BEs with Cas9 instead of its variants as the first choice for precise base editing of a target base, following the guidelines developed in one of our previous studies [34].

Recent studies have shown that the fusion of DNA‐binding proteins to base editors can significantly enhance editing efficiency by promoting the interaction between the deaminase and its DNA substrate [49, 58]. In this study, we systematically screened four distinct classes of DNA‐interacting proteins and successfully identified two proteins, HMGN1 and VP64, that notably increase editing efficiency (Figure 3). While the fusion of individual factors led to only a modest enhancement in efficiency (average increases of 1.17‐fold and 1.42‐fold for HMGN1 and VP64, respectively), we discovered that combining both factors in an optimal configuration resulted in a far more pronounced increase in base editing efficiency (average increase of 4.24‐fold; Figure 4; Supplementary Figure S14). This synergistic enhancement highlights the potential of integrating different types of DNA‐interacting factors into BE proteins to improve editing performance. Our findings thus provide valuable insights into optimization strategies for genome editing reagents.

The variance in efficiency enhancement observed between the CDA1 and rAPOBEC1 systems suggests that the potentiating effect of DNA‐binding domains is likely influenced by conformational constraints and deaminase‐specific compatibility with the fused protein(s). While we positioned the fusion modules at the N‐terminus to strictly maintain editing precision, other studies suggest that placing such domains between the deaminase and the Cas9 nickase might offer a higher efficiency boost [49]. Furthermore, a recent study demonstrates that certain DNA‐binding domains exhibit strong deaminase bias such as a Rad51 domain that significantly boosts rAPOBEC1‐based editors but fails to provide a similar enhancement for CDA1 systems [59]. Future structural optimization tailored to specific deaminases will therefore be valuable for maximizing the broader applications of this fusion strategy.

Although the HMGN1‐VP64 fusion significantly improved editing at NYN sites relative to controls, the absolute efficiency at these restrictive PAMs remains lower than at NRN sites, suggesting that the intrinsic SpRY–DNA binding affinity likely remains a rate‐limiting step. A promising strategy to address this limitation is the engineering of SpRY variants with enhanced affinity for NYN PAMs. For instance, recent studies have demonstrated that SpRYc, a chimeric variant incorporating the PAM‐interacting domain of Sc++ Cas9, exhibits markedly improved recognition of NYN PAMs compared to the original SpRY [60]. Notably, this modification translated to superior editing efficiency in human cells, indicating that integrating similar engineering strategies into our fusion architecture could further unlock the potential of high‐precision base editing at the most challenging genomic targets.

While PE offers unprecedented versatility for insertions, deletions, and all 12 types of base substitutions, it remains constrained by variable efficiency at certain genomic loci and complex design requirements. Our head‐to‐head comparison with plant‐optimized prime editors (ePPE) highlights a specific niche where our system provides a distinct advantage. Specifically, our data demonstrate that for generating precise C‐to‐T transitions, particularly at restrictive non‐NGG sites, our optimized high‐precision BEs significantly outperform ePPE in terms of editing frequency. The enhanced efficiency and PAM‐flexible targeting capability of our BEs address key limitations in plant biotechnology, opening up new possibilities for crop improvement by overcoming constraints associated with restrictive PAMs and low editing efficiency. We propose that our high‐precision BEs and PEs serve complementary roles in the precision editing toolbox: while PEs excel in introducing diverse edits with minimal bystander effects, our enhanced BEs provide superior efficiency for cytosine base editing and greater flexibility with respect to genomic targets. Ultimately, the choice between these tools should be guided by the specific requirements of the application, balancing the need for edit versatility and purity against the requirement for maximal editing efficiency.

## 4 Experimental Section

### 4.1 Yeast Strains and Growth Conditions

The diploid yeast strain *Saccharomyces cerevisiae* BY4743 (*MAT* a/α, *his3*Δ*1/his3*Δ*1, leu2*Δ*0/leu2*Δ*0, LYS2/lys2*Δ*0, met15*Δ*0/MET15, ura3*Δ*0/ura3*Δ) was used for all yeast genomic editing assays. Yeast propagation was performed in YPAD non‐selective liquid medium (containing 2% peptone, 1% yeast extract, 2% glucose, and 0.003% adenine hemisulfate), with 1.5% agar added for plate cultures. Positive colony selection after transformation was carried out on two‐deficiency medium SC‐L‐U (containing 0.67% YNB, 2% glucose, and a mixture of amino acids excluding uracil and leucine) with URA3 and LEU2 as selectable markers. Base editor expression was induced by culturing yeast in liquid medium containing 0.67% YNB, 2% galactose, 1% raffinose, and a mixture of amino acids excluding uracil and leucine, at 28°C with shaking at 225 rpm.

### 4.2 DNA Methods

PCR was performed using Phanta Max Super‐Fidelity DNA Polymerase (Vazyme Biotech) according to the manufacturer's instructions. All primers used in this study are listed in Supplementary Table S2. Cloning and plasmid amplification were carried out in *E. coli* strain DH5α. To generate CDA1‐SpG‐BE3 variants, six point mutations (D1135L/S1136W/G1218K/ E1219Q/R1335Q/T1337R) were introduced into nCas9 by PCR with primers containing the desired mutations. The resulting four fragments were cloned into the NruI/NcoI‐digested pJT45_GalL_nCDA1‐BE3 vector (Addgene plasmid #145038) to obtain CDA1‐SpG‐BE3 using the In‐Fusion HD Cloning Kit (Clontech). The CDA1‐SpRY‐BE3 variant was constructed by amplifying two DNA fragments containing the A61R mutation, which were then cloned into the SpeI/NdeI‐digested CDA1‐SpG‐BE3 vector. Subsequently, two additional fragments with four mutations (L1111R/N1317R/A1322R/R1333P) were ligated into the NruI/NcoI‐digested intermediate vector. To generate SpG or SpRY BEs with CDA1 truncations, DNA fragments encoding different truncated versions of CDA1 were PCR‐amplified from previously reported CDA1 BE plasmids harboring the various truncated versions [33, 34], and then ligated into SpeI/SbfI‐digested SpG‐BE3 or SpRY‐BE3 vectors. To construct factor‐CDA1Δ194‐SpRY‐BE3, an AvrII restriction site was introduced, and various DNA interaction factors were codon‐optimized for yeast and *de novo* synthesized (Tsingke Biotech). The synthetic gene fragments were then cloned into the AvrII‐digested CDA1Δ194‐SpRY‐BE3 vector using T4 DNA ligase (NEB). To generate plasmids expressing sgRNAs targeting specific sites (Supplementary Table S1), protospacer sequences were introduced through PCR amplification (as part of the primer sequence; see Supplementary Table S2). The resulting fragments were cloned into the AatII/KpnI‐digested vector pJT303_SNR52_sgRNA_Can1‐3 (Addgene plasmid #145066) using the In‐Fusion HD Cloning Kit (Clontech).

For base editing in rice, all DNA sequences, including CDA1, SpRY mutation, 2× UGI, and the HMGN1‐VP64 fusion, were codon‐optimized for rice and synthesized (Tsingke Biotech Co., Ltd., Beijing). To generate the CDA1‐SpRY‐BE4 and CDA1Δ194‐SpRY‐BE4 constructs for rice transformation, CDA1 and CDA1Δ194 fragments were PCR‐amplified and ligated into the pH‐A3A‐PBE vector (Addgene plasmid #119774). The resulting vectors were digested with MluI and SacI, and then combined with the domain containing the SpRY mutations and 2× UGI using the In‐Fusion HD Cloning Kit (Clontech). For construction of the Fusion‐CDA1Δ194‐SpRY‐BE4 vector, the HMGN1‐VP64 fusion was PCR‐amplified and ligated into the NcoI/SbfI‐digested CDA1Δ194‐SpRY‐BE4 vector. To facilitate visual screening for positive transgenic rice calli, the eGFP coding sequence was inserted into the PmeI sites of the three vectors.

To compare our BEs with established PEs, pegRNAs were designed using the online tool (http://www.plantgenomeediting.net/), and the corresponding DNA sequences were synthesized by Tsingke Biotech. The pegRNA‐encoding DNA fragments were then ligated into the unique BsaI restriction site within the ePPE vector (Addgene plasmid #183097). sgRNA spacer sequences were generated by annealing synthetic oligonucleotides and inserted into the BsaI site of the constructs via Golden Gate Assembly. Specific sgRNA spacer sequences are listed in Supplementary Table S1.

### 4.3 Yeast Transformation and Genomic DNA Extraction

Yeast cells were transformed following a previously described protocol [61]. Briefly, cells were initially grown on YPAD plates for 2 days. The freshly grown cells (25 µL for each transformation) were collected, washed with sterilized ddH_2_O, and incubated with 100 mM LiAc for 10 min. Subsequently, they were incubated at 42°C with plasmid DNA mixtures (containing 0.5–1 µg of plasmid DNA, 240 µL of 50% PEG3350, 36 µL of 1 M LiAc, 50 µL of 2 mg/mL carrier DNA, and 20 µL of ddH_2_O) for 1–3 h. Transgenic clones were selected on SC‐L‐U media and confirmed through PCR analyses. Yeast genomic DNA was isolated according to a published protocol [62]. PCR products were purified using the PCR Purification kit (Omega) and subsequently subjected to sequencing.

### 4.4 Galactose Induction of Base Editor Expression

Yeast cultures were initiated from 3–5 transformed colonies by suspending them in 3 mL SC‐L‐U medium containing 2% glucose, and grown until they reached the stationary phase. Samples of 0.8 mL were then pelleted, washed three times with sterile water to remove any residual glucose, and subsequently resuspended in 5 mL SC‐L‐U induction medium supplemented with 2% galactose and 1% raffinose to an OD_600_ of approximately 0.3. The cells were then incubated under shaking at 200 rpm for 20 h.

### 4.5 Can1 Mutagenesis

BE expression was galactose‐induced for 20 h, and the yeast cells were then plated on YPAD‐rich or SC medium plates lacking arginine but containing 60 µg/mL L‐canavanine (Sigma‐Aldrich). After incubation for 3 days, the number of colonies on each plate was recorded. The frequency of C‐to‐T mutation in *CAN1* was calculated by dividing the colony count on canavanine‐containing plates by the colony count on YPAD‐rich medium plates. Each experiment was performed at least three times on different days. To analyze the mutation spectrum, colonies were randomly chosen followed by PCR amplification of the targeted *CAN1* fragment and DNA sequencing. Control cultures that were not exposed to base editors did not yield any canavanine‐resistant colonies.

### 4.6 Rice Transformation

The BE constructs for rice were introduced into *Agrobacterium tumefaciens* strain EHA105 and transformed into embryogenic calli derived from mature seeds of the rice variety Ningjing 7 (*Oryza sativa* L. ssp. japonica). 3 days post‐transformation, the calli were transferred to N6 medium supplemented with 2.5 mg/L 2,4‐dichlorophenoxyacetic acid, 0.3 g/L casamino acids, 4.6 g/L Gelrite, 30 g/L sucrose, 0.25 g/L carbenicillin, and 75 mg/L hygromycin, and cultured for 30 days. GFP‐positive, hygromycin‐resistant calli were then collected and pooled for genomic DNA extraction, followed by genotyping using next‐generation sequencing (NGS).

### 4.7 High‐Throughput DNA Sequencing and Data Analysis

To prepare for high‐throughput sequencing in yeast, freshly transformed colonies were picked and grown in 3 mL SC‐L‐U medium with 2% glucose to stationary phase. 0.8 mL aliquots of each culture were then washed at least two times and resuspended in SC‐L‐U induction medium supplemented with 2% galactose and 1% raffinose to an OD_600_ ≈ 0.3, followed by growth for 20 h on a rotary shaker at 200 rpm at 28°C. Afterward, genomic DNA was extracted from 0.5 mL samples of each culture.

For high‐throughput sequencing, the regions targeted by base editing were PCR amplified using primer pairs containing index tags for multiplexing (Supplementary Table S2). PCR amplification was carried out with Phanta Max Super‐Fidelity DNA Polymerase (Vazyme Biotech), followed by purification of the amplified fragments with the NucleoSpin Gel and PCR clean‐up kit (Omega). The resulting index‐labeled PCR products were pooled at equal molar ratios, and PCR‐free library construction, high‐throughput sequencing, demultiplexing, and data processing were performed commercially (Bokaisen Biotech Ltd.). Paired‐end sequencing was performed on an Illumina NovaSeq 6000 platform to obtain 150 nt read length for each side, with an average of more than 100 000 reads obtained for each sample. Clean FASTQ files obtained after data filtering were analyzed using custom‐written scripts (available at https://github.com/zfcarpe/Cas9Sequencing).

### 4.8 Whole‐Genome Sequencing for Analysis of Off‐Target Editing

Yeast strains expressing various base editor constructs, including CDA1‐BE3, CDA1‐SpRY‐BE3, and three truncated versions, along with sgRNAs targeting the *Can1* locus were initially grown in SC‐L‐U medium containing 2% glucose. Afterward, the cells were transferred to induction medium supplemented with 2% galactose and 1% raffinose, and incubated for 20 h. Following induction, appropriate volumes of each culture were plated on YPAD or SC‐Arg medium supplemented with 60 µg/mL L‐canavanine (Sigma‐Aldrich) and allowed to grow for 3 days to select for resistant colonies. Three colonies were randomly selected from each plate, suspended in YPAD medium, and incubated overnight. The resulting cultures were then mixed at equal volumes and genomic DNA was extracted using the Genomic DNA Purification Kit (Solarbio, China) following the manufacturer's instructions. The quality of the extracted DNA samples was assessed, and commercially available services were used for library construction, high‐throughput sequencing, and bioinformatic analysis of the raw data (Bokaisen Biotech Ltd.). DNA sequencing was performed on an Illumina NovaSeq 6000 platform. This sequencing generated approximately 1 Gb of clean data per sample, yielding an average sequencing depth of 83×.

## Author Contributions

J. T. conceived the project. J. T. and X. L. designed the experiments. X. L., Y. Q., Z. Y., Z. L., Y. Z., L. L., S. L., and W. Z. performed the experiments. X. L. performed bioinformatic analyses. J. T. and X. L. wrote the manuscript. R. B., J. W., and M. W. critically commented on and edited the manuscript. All authors contributed to data analysis, and edited and approved the manuscript.

## Conflicts of Interest

The authors declare no competing financial interests.

## Supporting information




**Supporting File 1**: advs76047‐sup‐0001‐SuppMat.docx.

**Supporting File 2**: advs76047‐sup‐0002‐Table.xlsx.

**Supporting File 3**: advs76047‐sup‐0003‐DataFiles.xlsx.

## Data Availability

The data supporting the findings of this study are available within the paper and its Supporting Information files. High‐throughput sequencing data have been deposited in the National Center for Biotechnology Information Sequence Read Archive database under accession code PRJNA1219349. The source data underlying Figures 1, 2, 3, 4, 5, 6, and Supplementary Figures S5–S17 are provided as a Source Data file. Python scripts used in this study are available at GitHub (https://github.com/zfcarpe/Cas9Sequencing).
