# Supplementary material for: Synergistic HMGN1 and VP64 Fusions Potentiate High‐Precision and PAM‐Flexible Base Editing
Source: Adv Sci (Weinh). 2026 Jun 11:e76047. Online ahead of print. doi: 10.1002/advs.76047 (PMC13336815; doi:10.1002/advs.76047)
Supplement: Supplementary file 1 — Supporting File 1: advs76047‐sup‐0001‐SuppMat.docx. [file ADVS-9999-e76047-s001.docx]

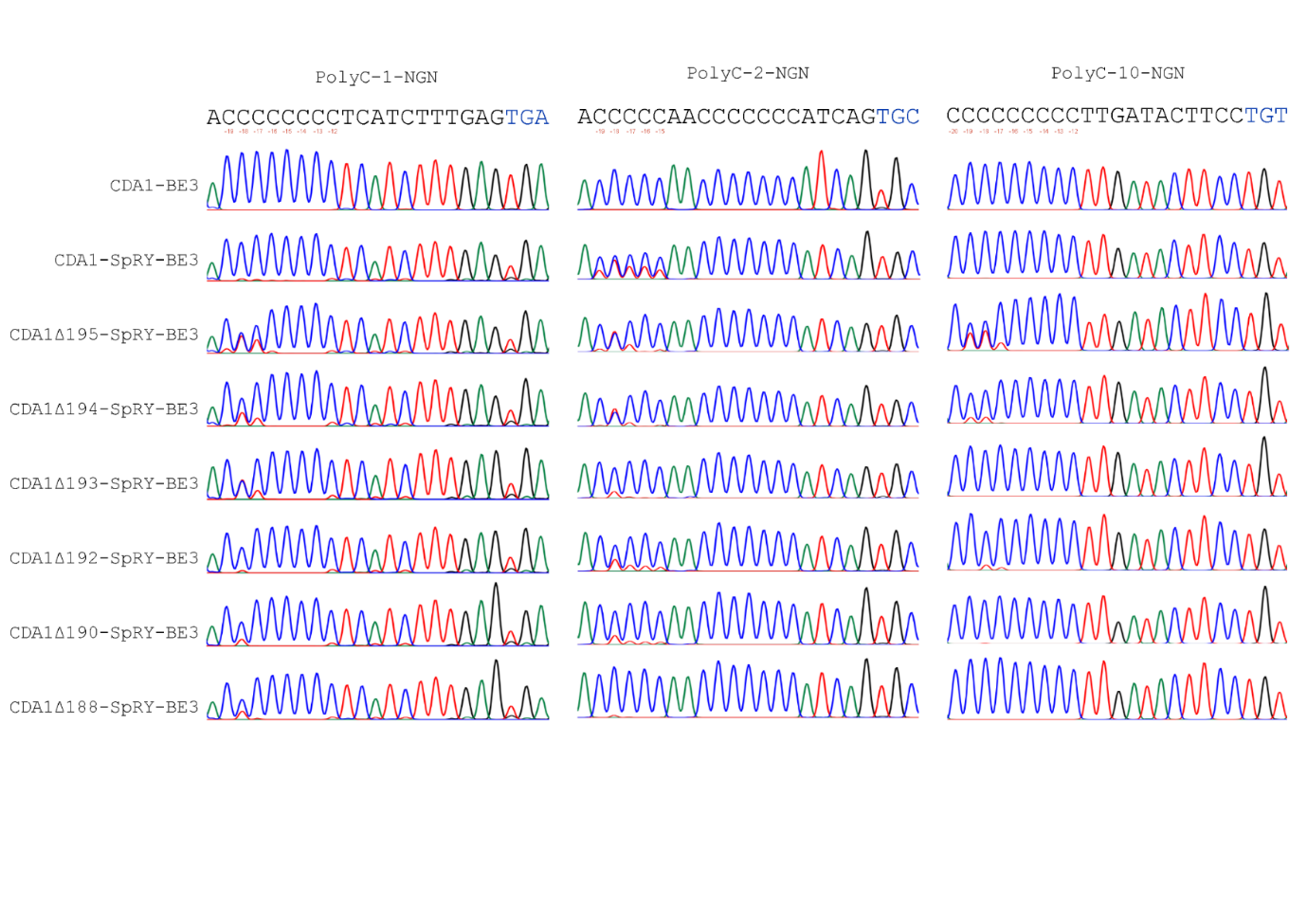


**Supplementary Figure 1** Analysis of base editing by a set of SpRY BEs fused to the full-length CDA1 (CDA1-SpRY-BE3) or C-terminally truncated versions of CDA1 (CDA1Δ195-SpRY-BE3; CDA1Δ194-SpRY-BE3; CDA1Δ193-SpRY-BE3; CDA1Δ192-SpRY-BE3; CDA1Δ190-SpRY-BE3; CDA1Δ188-SpRY-BE3; cf. Fig. 1). The three tested target sequences (PolyC-1-NGN, PolyC-2-NGN and PolyC-10-NGN) all contain an oligo(C) stretch upstream of the PAM sequence NGN that is recognized by SpRY. The amplified target sequences were directly sequenced by the Sanger method, and the sequence chromatograms are shown for each BE. The nucleotide sequences are shown above the chromatograms, with the PAM indicated in blue and the cytidines in the oligo(C) stretch marked in red (numbered relative to the PAM). The reciprocal full-length fusion, CDA1-BE3, was also analyzed. CDA1-BE3 has no PAM in proper distance from the oligo(C) stretch and served as negative control.


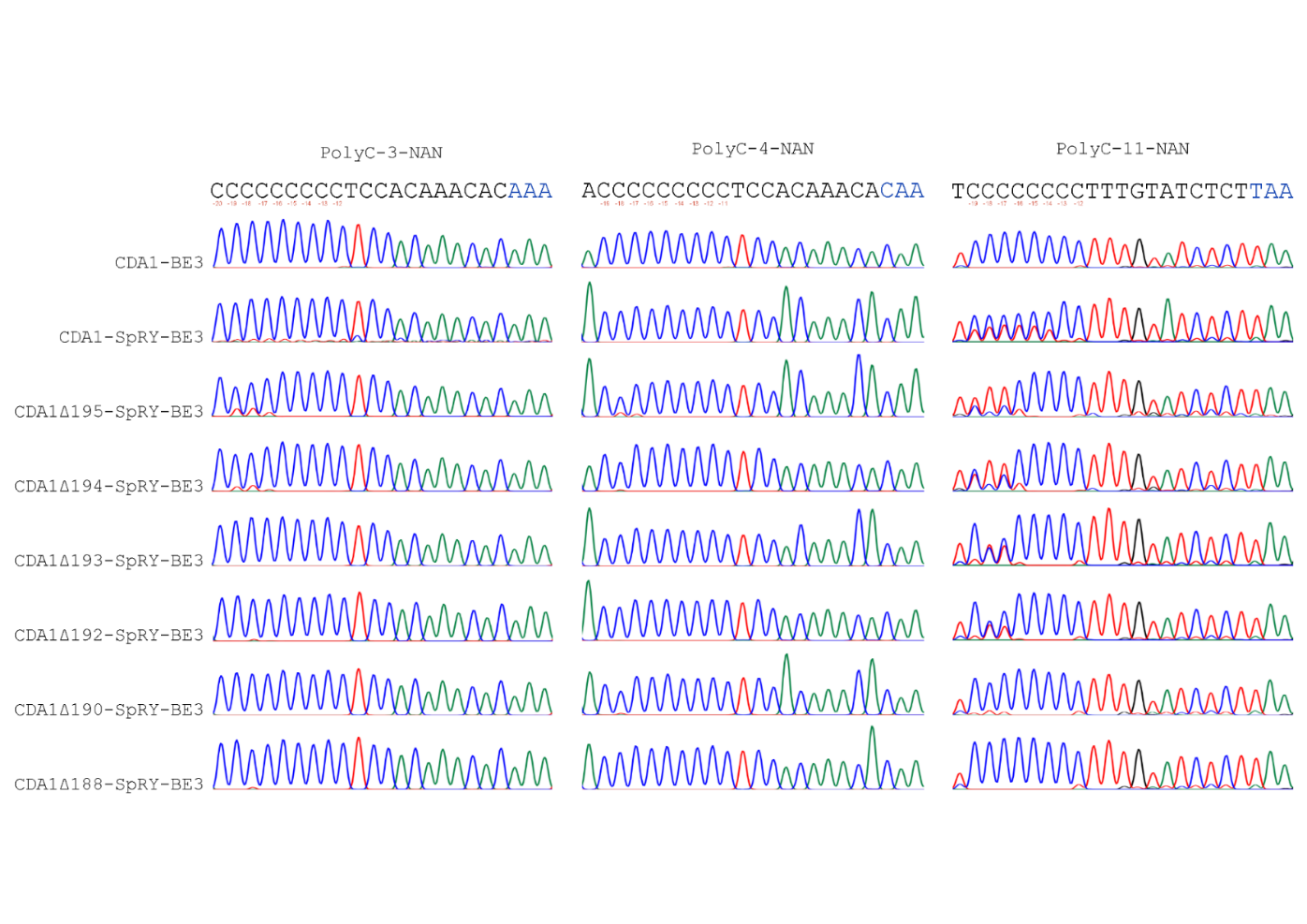


**Supplementary Figure 2** Analysis of base editing by a set of SpRY BEs fused to the full-length CDA1 (CDA1-SpRY-BE3) or C-terminally truncated versions of CDA1 (CDA1Δ195-SpRY-BE3; CDA1Δ194-SpRY-BE3; CDA1Δ193-SpRY-BE3; CDA1Δ192-SpRY-BE3; CDA1Δ190-SpRY-BE3; CDA1Δ188-SpRY-BE3; cf. Fig. 1). The three tested target sequences (PolyC-3-NAN, PolyC-4-NAN and PolyC-11-NAN) all contain an oligo(C) stretch upstream of the PAM sequence NAN that is recognized by SpRY. The amplified target sequences were directly sequenced by the Sanger method, and the sequence chromatograms are shown for each BE. The nucleotide sequences are shown above the chromatograms, with the PAM indicated in blue and the cytidines in the oligo(C) stretch marked in red (numbered relative to the PAM). The reciprocal full-length fusion, CDA1-BE3, was also analyzed. CDA1-BE3 has no PAM in proper distance from the oligo(C) stretch and served as negative control.


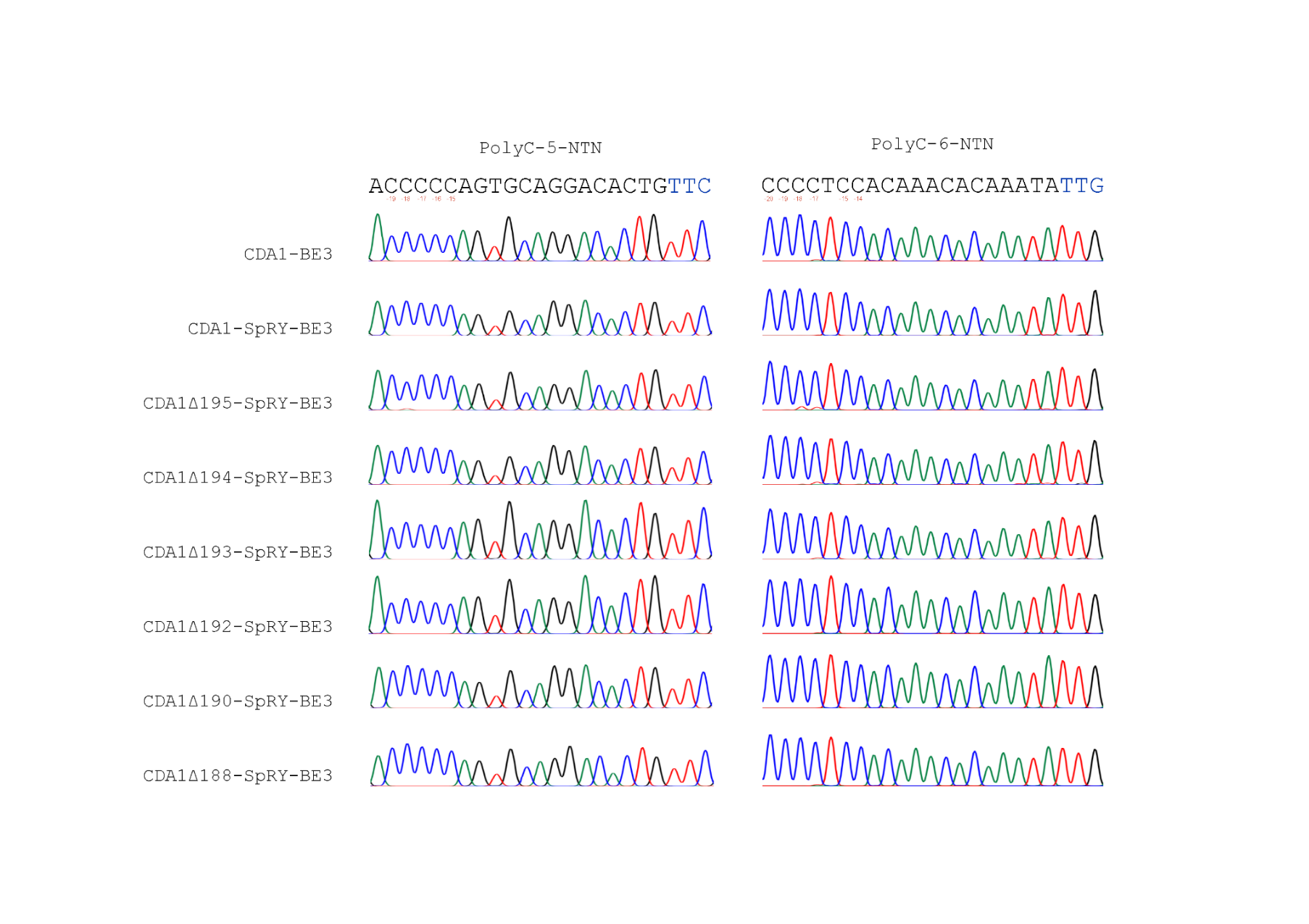


**Supplementary Figure 3** Analysis of base editing by a set of SpRY BEs fused to the full-length CDA1 (CDA1-SpRY-BE3) or C-terminally truncated versions of CDA1 (CDA1Δ195-SpRY-BE3; CDA1Δ194-SpRY-BE3; CDA1Δ193-SpRY-BE3; CDA1Δ192-SpRY-BE3; CDA1Δ190-SpRY-BE3; CDA1Δ188-SpRY-BE3; cf. Fig. 1). The two tested target sequences (PolyC-5-NTN and PolyC-6-NTN) all contain an oligo(C) stretch upstream of the PAM sequence NTN that is recognized by SpRY. The amplified target sequences were directly sequenced by the Sanger method, and the sequence chromatograms are shown for each BE. The nucleotide sequences are shown above the chromatograms, with the PAM indicated in blue and the cytidines in the oligo(C) stretch marked in red (numbered relative to the PAM). The reciprocal full-length fusion, CDA1-BE3, was also analyzed. CDA1-BE3 has no PAM in proper distance from the oligo(C) stretch and served as negative control.


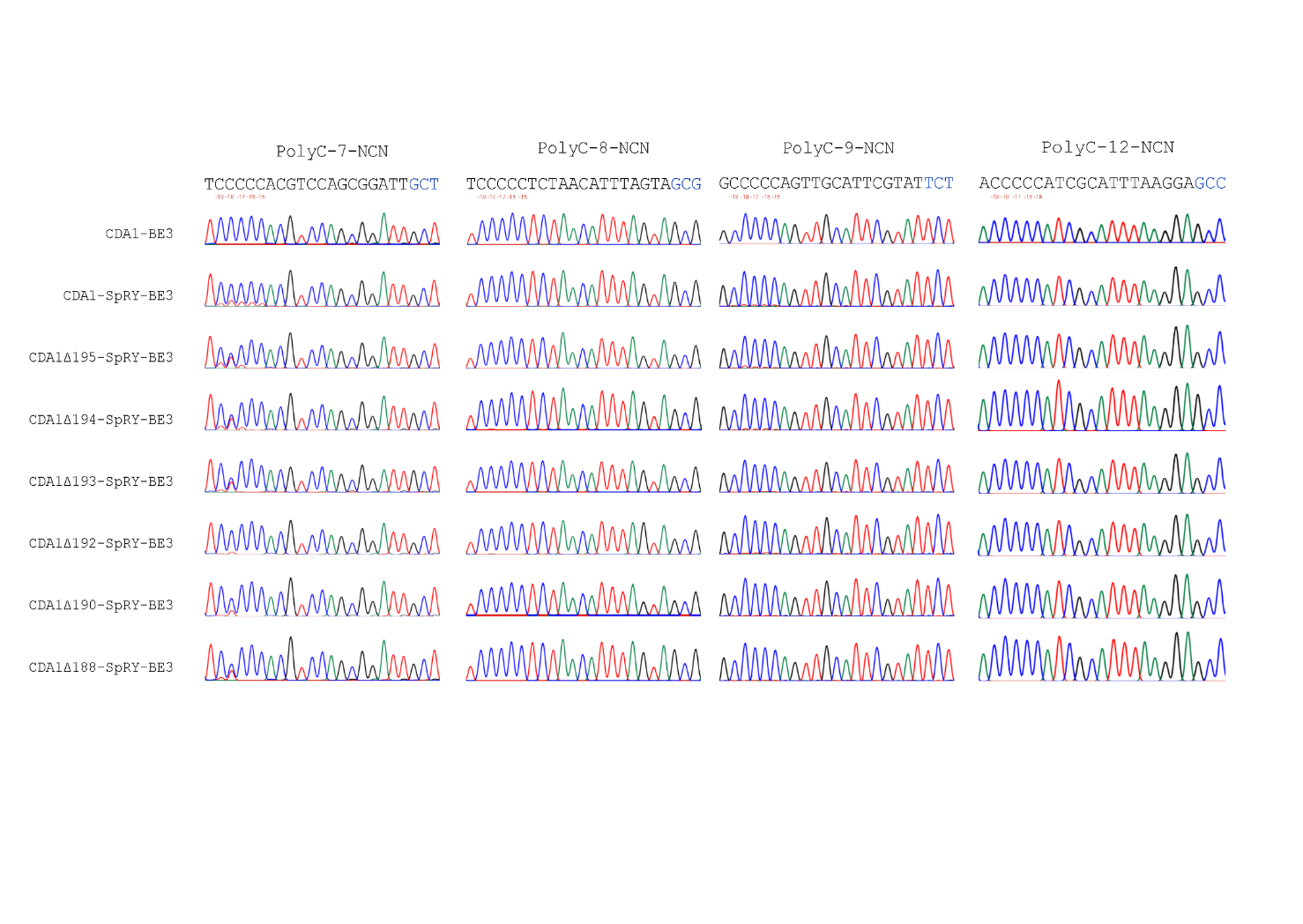


**Supplementary Figure 4** Analysis of base editing by a set of SpRY BEs fused to the full-length CDA1 (CDA1-SpRY-BE3) or C-terminally truncated versions of CDA1 (CDA1Δ195-SpRY-BE3; CDA1Δ194-SpRY-BE3; CDA1Δ193-SpRY-BE3; CDA1Δ192-SpRY-BE3; CDA1Δ190-SpRY-BE3; CDA1Δ188-SpRY-BE3; cf. Fig. 1). The four tested target sequences (PolyC-7-NCN, PolyC-8-NCN, PolyC-9-NCN and PolyC-12-NCN) all contain an oligo(C) stretch upstream of the PAM sequence NCN that is recognized by SpRY. The amplified target sequences were directly sequenced by the Sanger method, and the sequence chromatograms are shown for each BE. The nucleotide sequences are shown above the chromatograms, with the PAM indicated in blue and the cytidines in the oligo(C) stretch marked in red (numbered relative to the PAM). The reciprocal full-length fusion, CDA1-BE3, was also analyzed. CDA1-BE3 has no PAM in proper distance from the oligo(C) stretch and served as negative control.


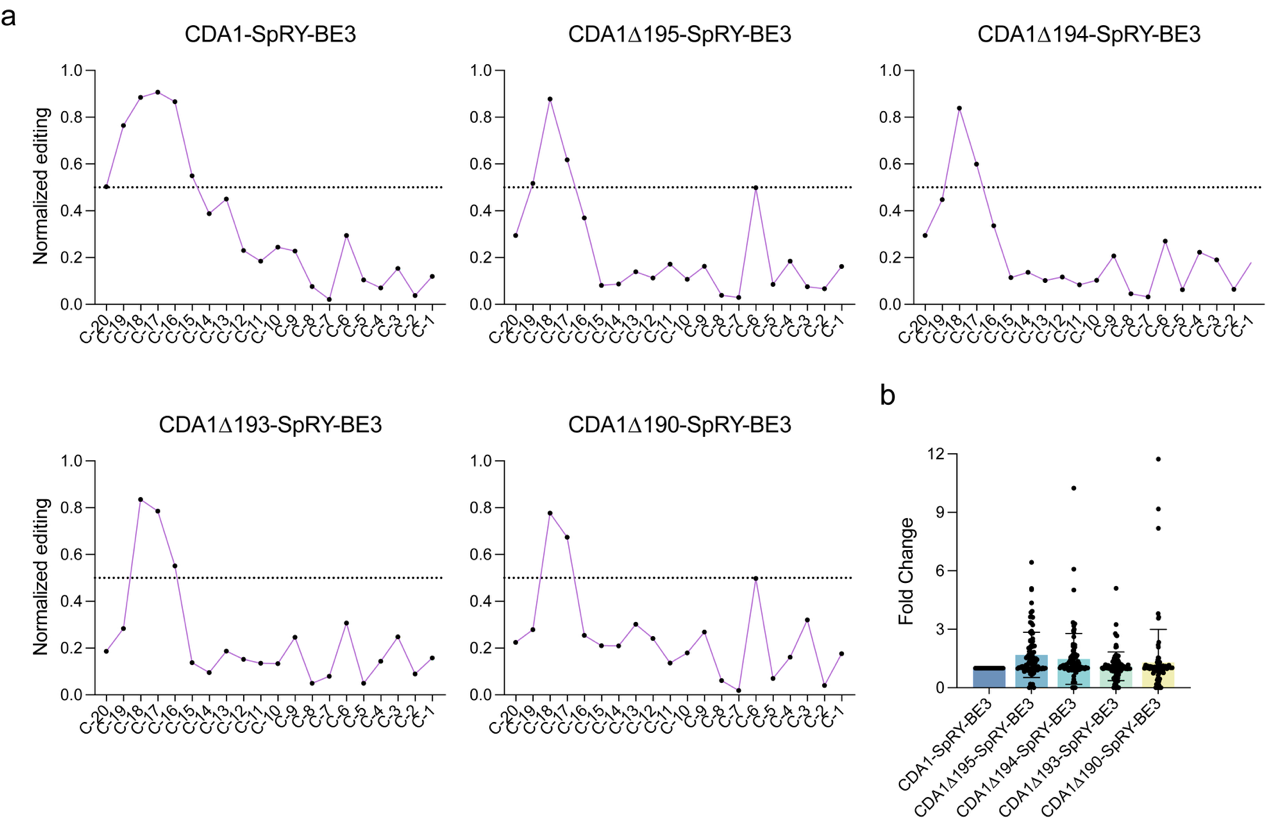


**Supplementary Figure 5.** Evaluation of editing precision and efficiency across CDA1-SpRY-BE3, CDA1∆195-SpRY-BE3, CDA1∆194-SpRY-BE3, CDA1∆193-SpRY-BE3, and CDA1∆190-SpRY-BE3. **a** Base editing window profiles for the canonical untruncated editor and the tested truncations. The horizontal dashed line indicates a relative editing efficiency of 0.5, and each black dot represents the mean relative editing efficiency at the corresponding position. Normalized C-to-T editing was calculated by normalizing the editing efficiency of each C position to the C position with the highest editing activity within the same target site. **b** Quantitative fold change in average editing efficiency mediated by each editor across multiple target sites. The editing efficiency mediated by CDA1-SpRY-BE3 at each target site was used as the baseline reference and set to 1. The black dots represent individual targets (n=86), and the error bars represent the mean ± SD. Source data are provided as a Source Data file.


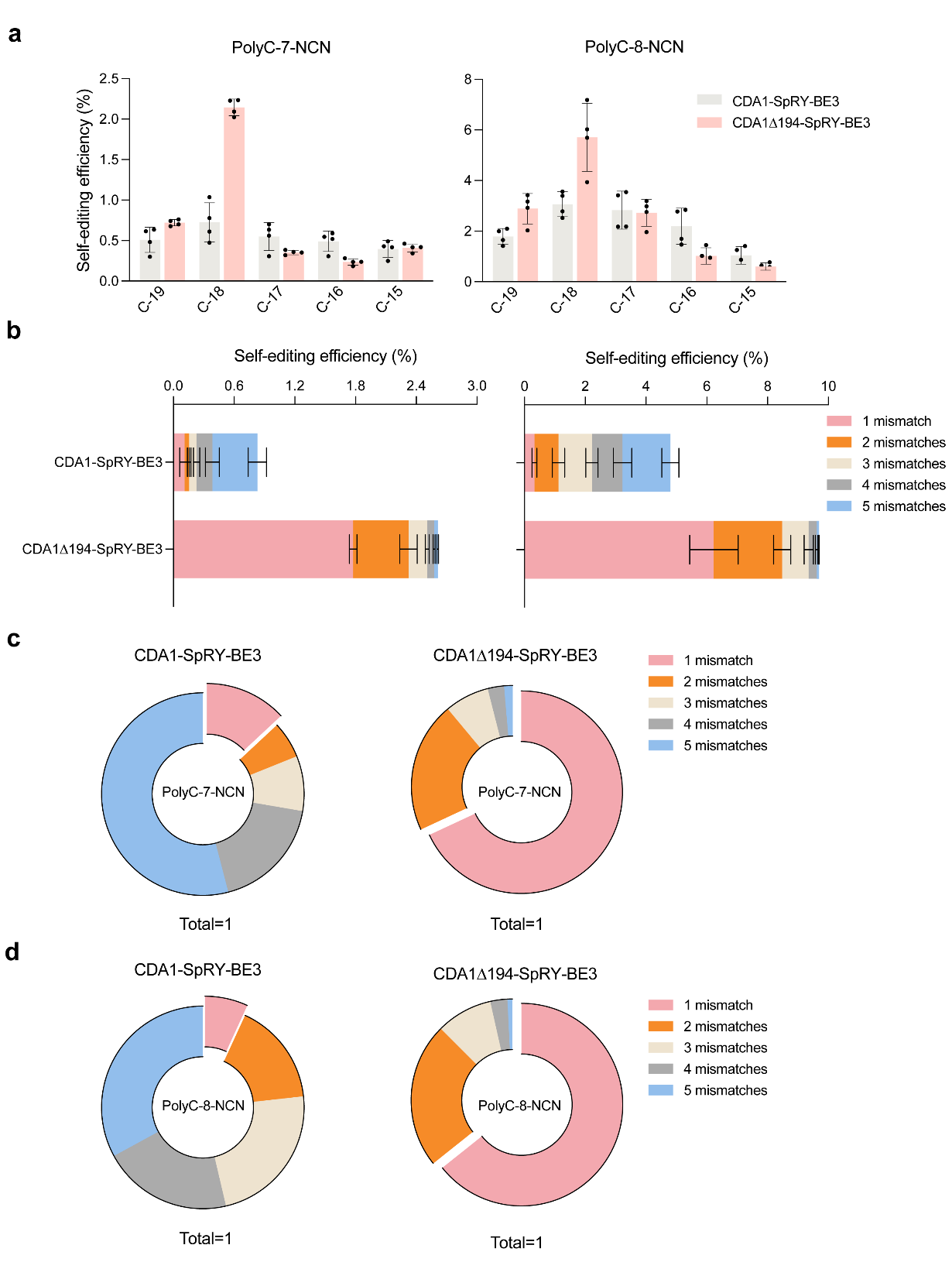


**Supplementary Figure 6.** Self-editing effects mediated by CDA1-SpRY-BE3 and CDA1Δ194-SpRY-BE3 at the PolyC-7-NCN and PolyC-8-NCN sites. **a** Self-editing efficiencies at individual cytosine positions induced by CDA1-SpRY-BE3 and CDA1Δ194-SpRY-BE3 at the PolyC-7-NCN and PolyC-8-NCN sgRNA spacer sequences. The data for the untruncated canonical editor and the truncated variant are represented by gray and pink bars, respectively. Self-editing efficiency is calculated as the percentage of C-to-T reads among the total reads at each corresponding cytosine position. Values and error bars represent the mean and standard deviation of four independent biological replicates, with individual data points shown as black dots. **b** Quantification of sgRNA spacer reads containing different numbers of C-to-T substitutions. Individual sequencing reads were classified according to the number of edited cytosines within the sgRNA spacer, corresponding to one, two, three, four, or five mismatches. Stacked bars show the mean percentages of total sgRNA reads carrying each mismatch class for CDA1-SpRY-BE3 and CDA1Δ194-SpRY-BE3 at the PolyC-7-NCN and PolyC-8-NCN sites. Error bars indicate the standard deviation of four independent biological replicates. **c-d** Relative composition of the self-edited sgRNA populations generated by CDA1-SpRY-BE3 and CDA1Δ194-SpRY-BE3 at the PolyC-7-NCN **(c)** and PolyC-8-NCN **(d)** sites. Donut plots show the normalized proportions of edited sgRNA reads containing one, two, three, four, or five mismatches, with the total edited sgRNA population set to 1 for each editor. Source data are provided as a Source Data file.


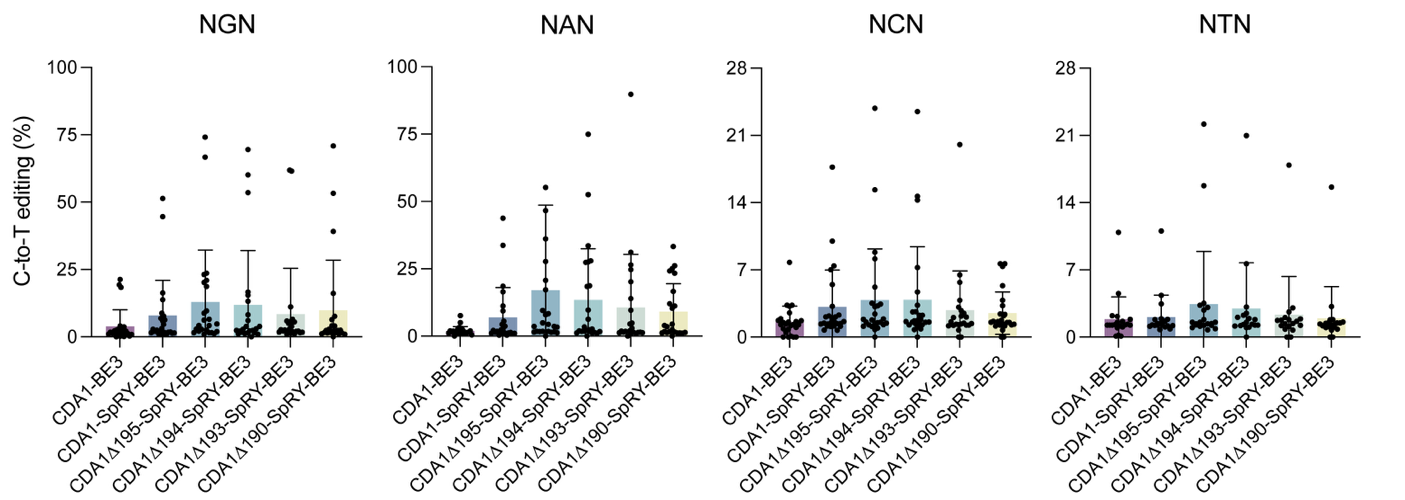


**Supplementary Figure 7.** Comparative analysis of editing efficiencies across diverse PAM categories. The bar charts display the C-to-T editing efficiency of various base editors on targets with NGN, NAN, NCN, and NTN PAMs. In each panel, the tested variants from left to right are CDA1-BE3, CDA1-SpRY-BE3, CDA1∆195-SpRY-BE3, CDA1∆194-SpRY-BE3, CDA1∆193-SpRY-BE3, and CDA1∆190-SpRY-BE3. The base editing results mediated by these six editors are represented by purple, dark blue, gray-blue, light blue, gray-green, and yellow bars respectively. The height of each bar represents the average editing efficiency mediated by the corresponding editor at multiple sites with the corresponding PAM sequences. The black dots represent individual targets (n>20), and the error bars indicate the mean ± SD. Source data are provided as a Source Data file.


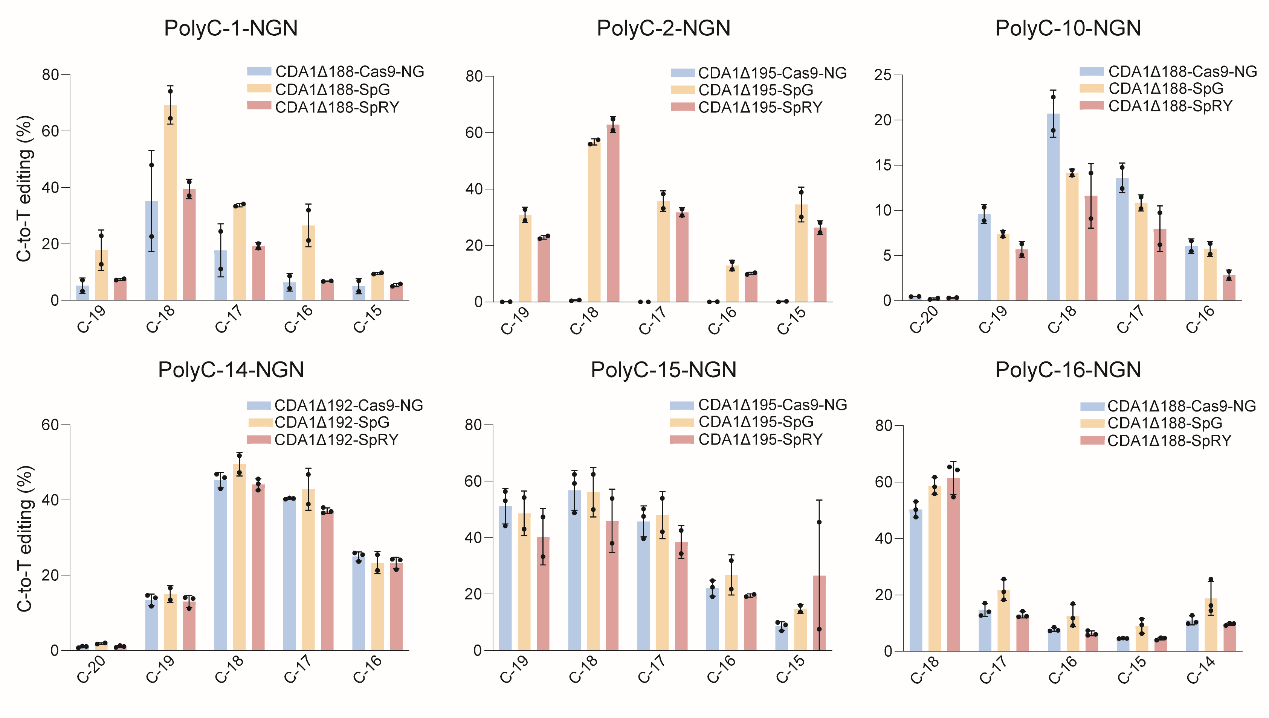


**Supplementary Figure 8** Comparison of base editing efficiency in targets with an NGN PAM sequence. PolyC-1-NGN, PolyC-2-NGN, PolyC-10-NGN, PolyC-14-NGN, PolyC-15-NGN, and PolyC-16-NGN all contain an oligo(C) stretch upstream of the PAM sequence, which can be recognized by Cas9-NG, SpG, and SpRY. The C-to-T conversion rates at each C were determined by NGS. Editing mediated by CDA1Δ-SpRY-BEs, CDA1Δ-SpG-BEs, and CDA1Δ-Cas9-NG-BEs are represented as red, yellow, and blue bars, respectively. Values and error bars represent the mean and standard deviation of at least two independent biological replicates. Source data are provided as a Source Data file.


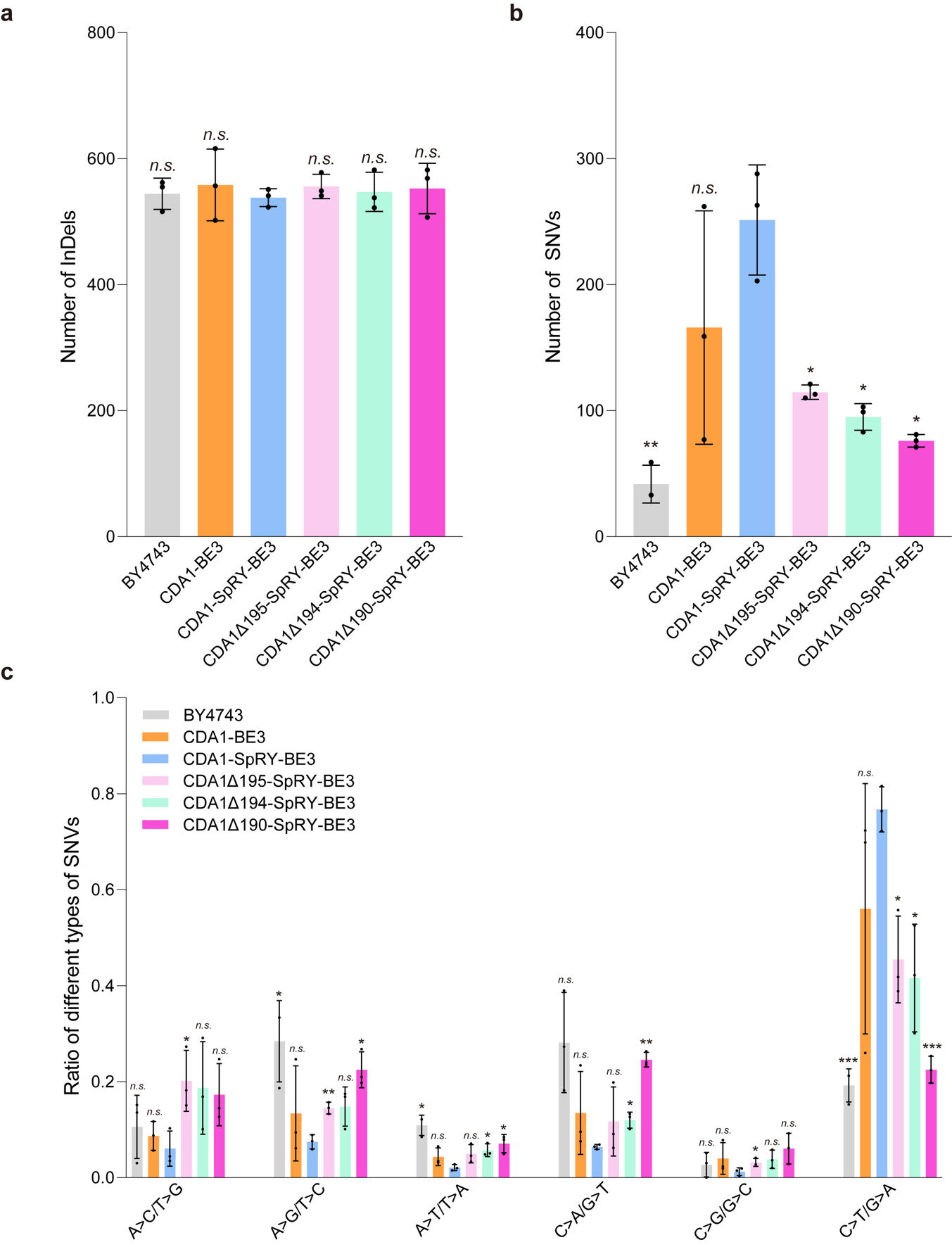


**Supplementary Figure 9** Analysis of off-target editing. Whole-genome sequencing was performed to identify genetic changes in strains containing CDA1-BE3, CDA1-SpRY-BE3, CDA1Δ195-SpRY-BE3, CDA1Δ194-SpRY-BE3, CDA1Δ190-SpRY-BE3, and a non-BE control. **a-b** Comparison of the total number of detected indels (**a**) and SNVs (**b**). **c** Mutation frequencies of different types of SNVs in cells treated with the five base editors and the non-BE control. The sgRNA was designed to target site *Can1* (Supplementary Table 1). Values and error bars represent the mean and standard deviation of three independent biological replicates. *P < 0.05, **P < 0.01, *n.s.,* not significant; CDA1-SpRY-BE3 was used as the control (Student's *t*-test). Source data are provided as a Source Data file.


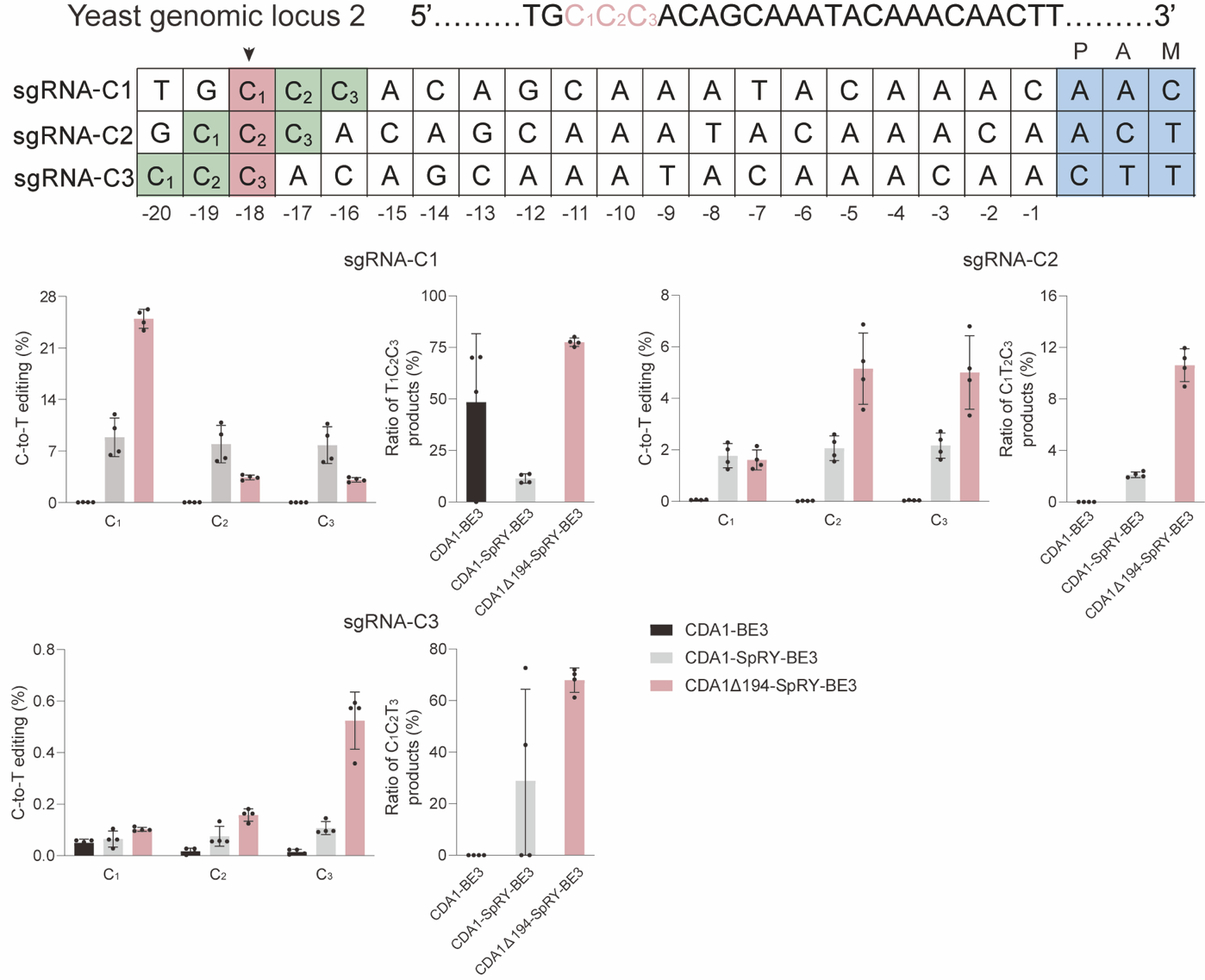


**Supplementary Figure 10** Test for precise base editing at yeast endogenous genomic locus 2. The target cytidines are labeled as C_1_, C_2_, and C_3_ from 5' to 3'. Each cytidine was individually targeted by sgRNA-mediated base editing (target C highlighted in red), with the other two Cs serving as bystander bases (highlighted in green). This design enabled testing of the ability of CDA1-truncated base editors to distinguish between multiple cytidines. To edit each target cytidine, a corresponding sgRNA was designed with the target C positioned at position -18. Three distinct sgRNAs were designed, named sgRNA-C_1_, sgRNA-C_2_, and sgRNA-C_3_, and their PAM sequences are highlighted in blue (for spacer sequences, see Supplementary Table 1, Group 2). Base editing outcomes for the three cytidines were evaluated by next-generation sequencing, and the fraction of desired editing products for each sgRNA was analyzed. Values and error bars represent the mean and standard deviation of four independent biological replicates. Source data are provided as a Source Data file.


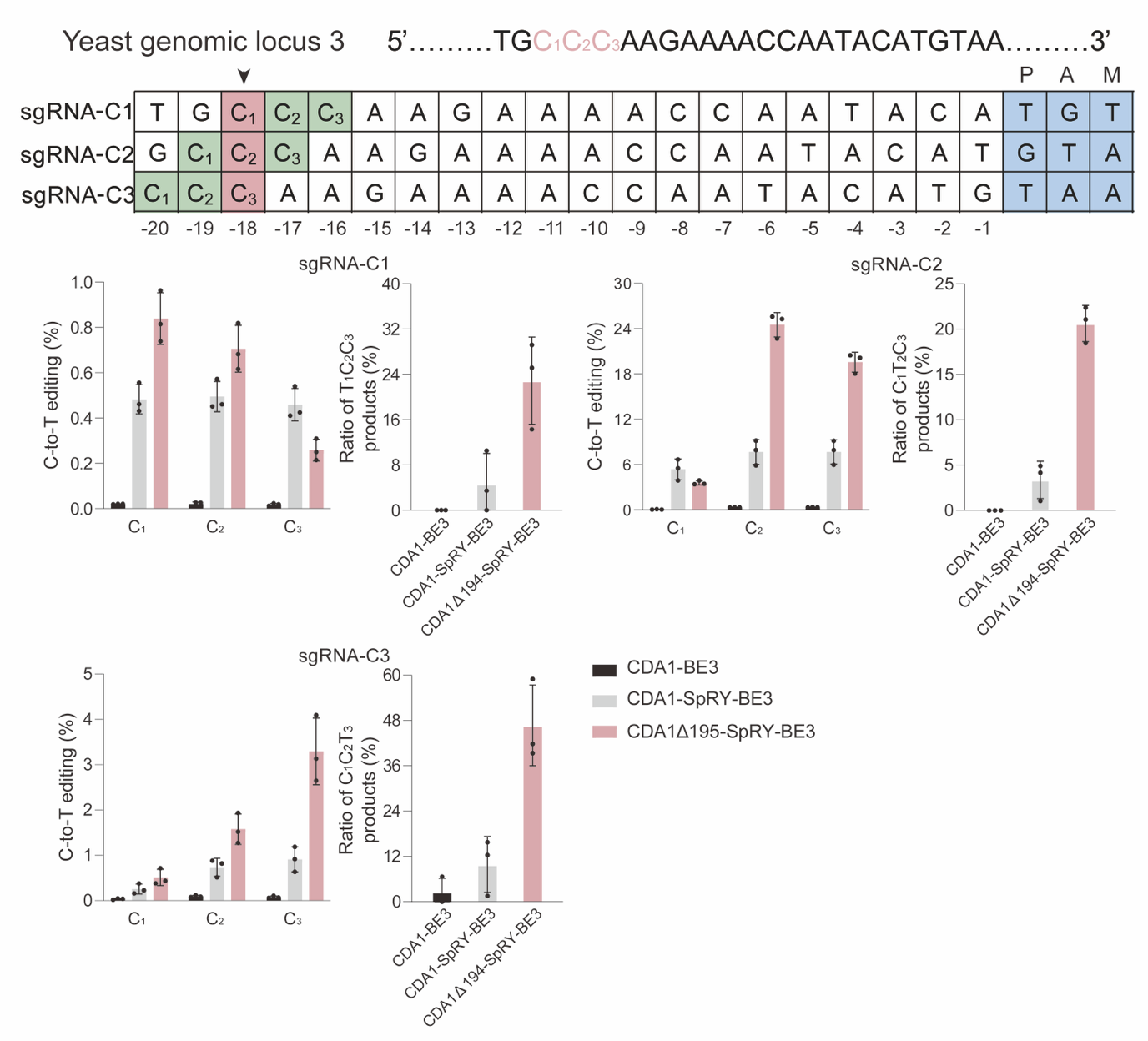


**Supplementary Figure 11** Test for precise base editing at yeast endogenous genomic locus 3. The target cytidines are labeled as C_1_, C_2_, and C_3_ from 5' to 3'. Each cytidine was individually targeted by sgRNA-mediated base editing (target C highlighted in red), with the other two Cs serving as bystander bases (highlighted in green). This design enabled testing of the ability of CDA1-truncated base editors to distinguish between multiple cytidines. To edit each target cytidine, a corresponding sgRNA was designed with the target C positioned at position -18. Three distinct sgRNAs were designed, named sgRNA-C_1_, sgRNA-C_2_, and sgRNA-C_3_, and their PAM sequences are highlighted in blue (for spacer sequences, see Supplementary Table 1, Group 3). Base editing outcomes for the three cytidines were evaluated by next-generation sequencing, and the fraction of desired editing products for each sgRNA was analyzed. Values and error bars represent the mean and standard deviation of three independent biological replicates. Source data are provided as a Source Data file.


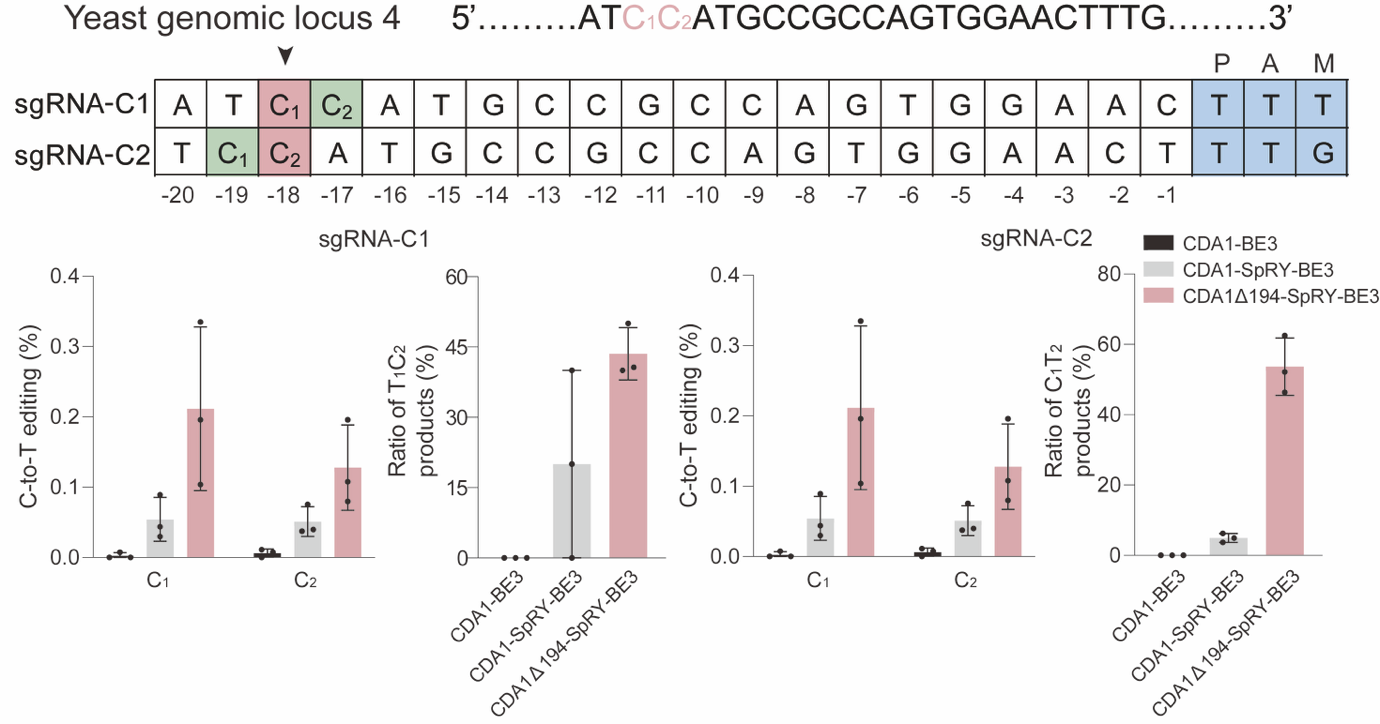


**Supplementary Figure 12** Test for precise base editing at yeast endogenous genomic locus 4. The target cytidines are labeled as C_1_ and C_2_ from 5' to 3'. Each cytidine was individually targeted by sgRNA-mediated base editing (target C highlighted in red), with the other C serving as bystander bases (highlighted in green). This design enabled testing of the ability of CDA1-truncated base editors to distinguish between multiple cytidines. To edit each target cytidine, a corresponding sgRNA was designed with the target C positioned at position -18. Two distinct sgRNAs were designed, named sgRNA-C_1_ and sgRNA-C_2_, and their PAM sequences are highlighted in blue (for spacer sequences, see Supplementary Table 1, Group 4). Base editing outcomes for the two cytidines were evaluated by next-generation sequencing, and the fraction of desired editing products for each sgRNA was analyzed. Values and error bars represent the mean and standard deviation of three independent biological replicates. Source data are provided as a Source Data file.


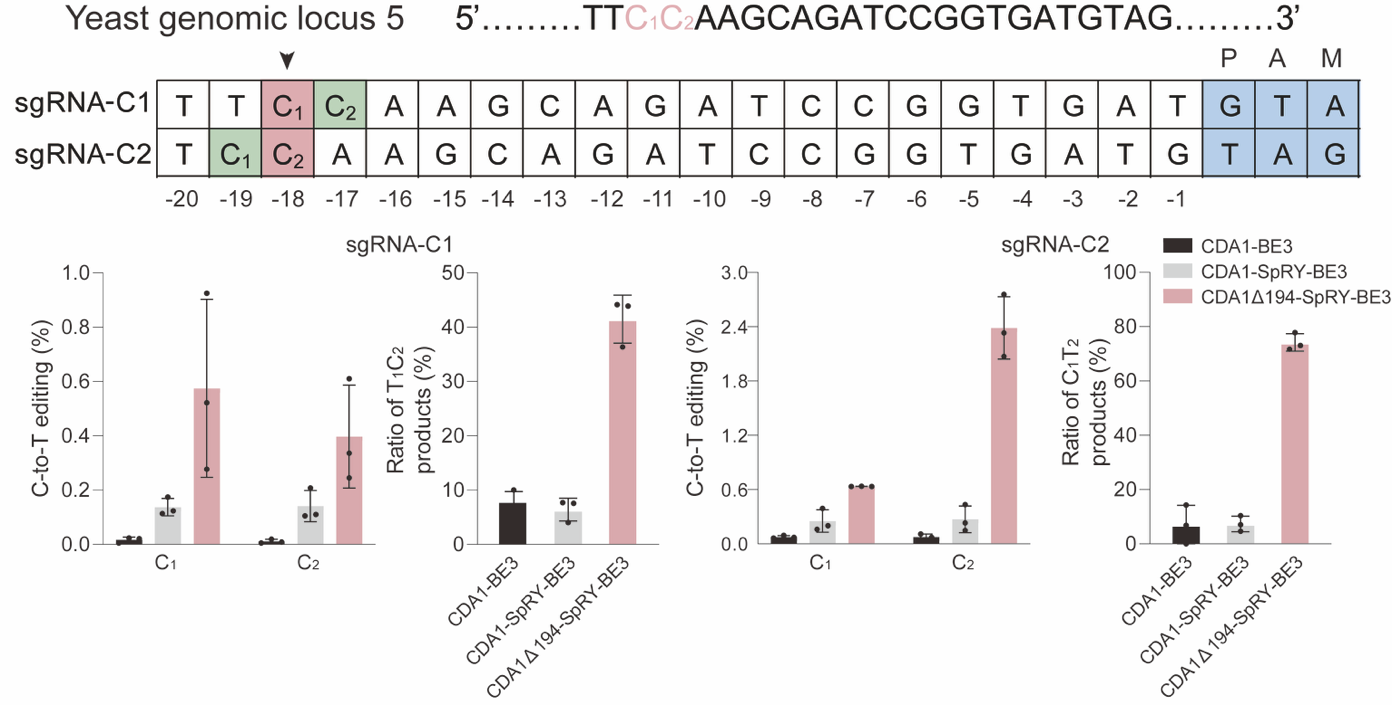


**Supplementary Figure 13** Test for precise base editing at yeast endogenous genomic locus 5. The target cytidines are labeled as C_1_ and C_2_ from 5' to 3'. Each cytidine was individually targeted by sgRNA-mediated base editing (target C highlighted in red), with the other C serving as bystander bases (highlighted in green). This design enabled testing of the ability of CDA1-truncated base editors to distinguish between multiple cytidines. To edit each target cytidine, a corresponding sgRNA was designed with the target C positioned at position -18. Two distinct sgRNAs were designed, named sgRNA-C_1_ and sgRNA-C_2_, and their PAM sequences are highlighted in blue (for spacer sequences, see Supplementary Table 1, Group 5). Base editing outcomes for the two cytidines were evaluated by next-generation sequencing, and the fraction of desired editing products for each sgRNA was analyzed. Values and error bars represent the mean and standard deviation of three independent biological replicates. Source data are provided as a Source Data file.


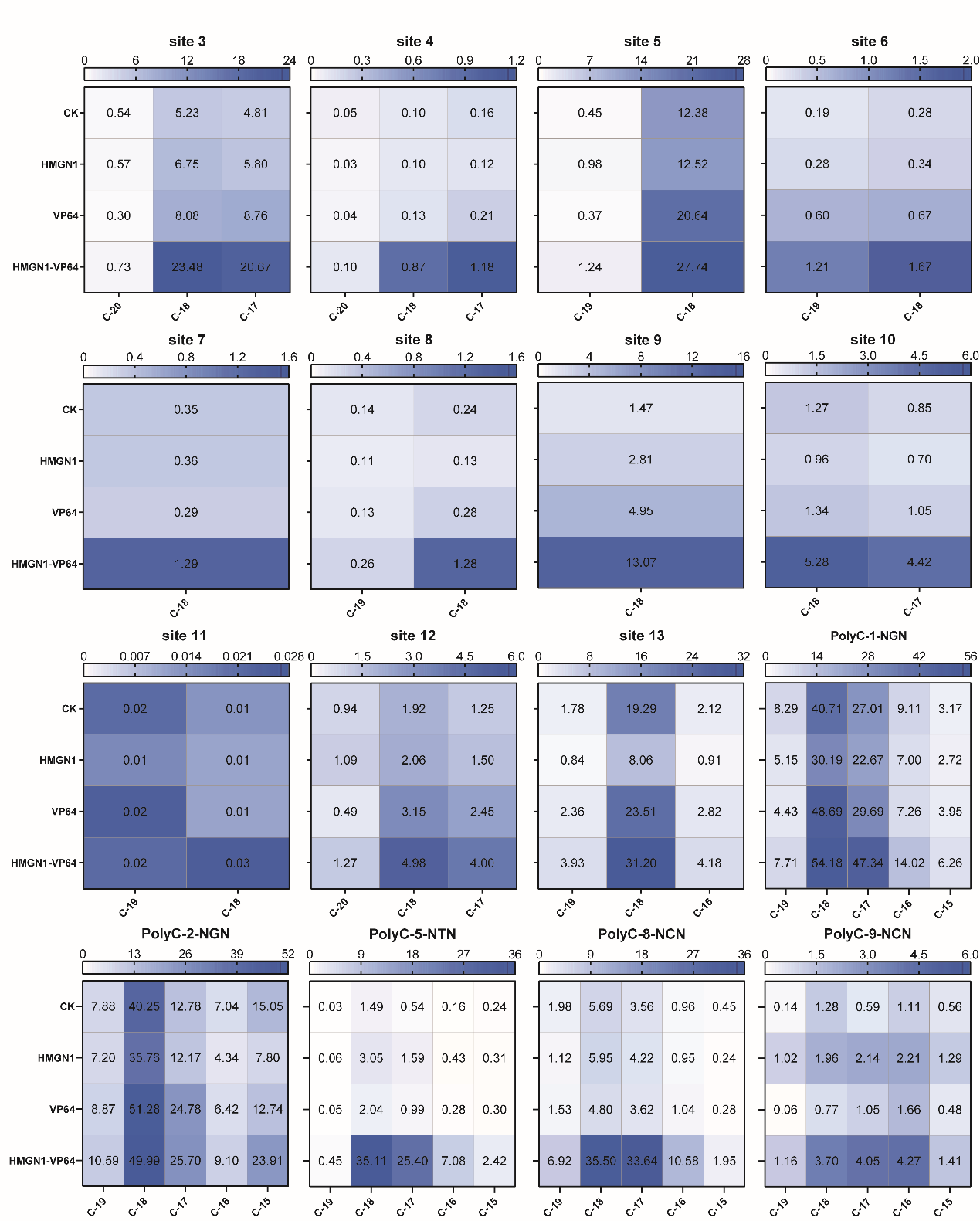


**Supplementary Figure 14** Evaluation of the effect of HMGN1 and VP64 fusions on base editing efficiency at sixteen different target sites. Heatmaps display C-to-T editing efficiencies at the cytosine positions in the sixteen target sites. For each target site, constructs harboring CK (control, CDA1Δ194-SpRY-BE3 alone), HMGN1, VP64, or the combination HMGN1-VP64 were tested. Editing efficiencies are visualized in shades of blue, with deeper tones representing higher editing efficiencies. Values inside each cell indicate the mean editing frequency (in %) of three independent biological replicates at the corresponding cytosine position. Source data are provided as a Source Data file.


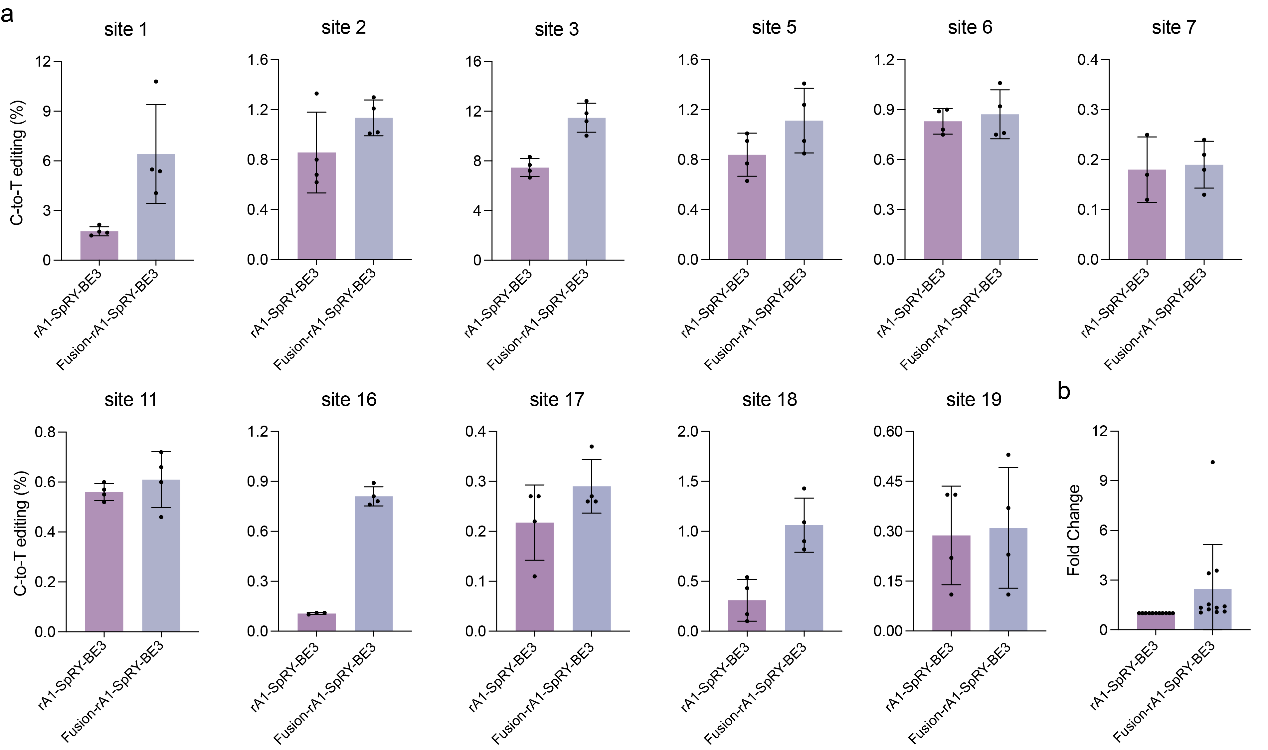


**Supplementary Figure 15**. Evaluation of C-to-T editing enhanced by the HMGN1-VP64 fusion to rA1-SpRY-BE3. **a** Targeted editing activities mediated by the basal rA1-SpRY-BE3 and the Fusion-rA1-SpRY-BE3 are shown for eleven independent genomic loci. The y-axis represents the proportion of reads with C-to-T conversion at the target site among total reads. The black dots represent individual data points from four independent biological replicates. **b** The quantitative fold change in Fusion-rA1-SpRY-BE3-mediated editing efficiency relative to the basal rA1-SpRY-BE3 across all 11 tested target sites. Each black dot in this panel represents the mean fold change for a distinct target site calculated from four independent biological replicates. The error bars in both panels represent the mean ± standard deviation. Source data are provided as a Source Data file.


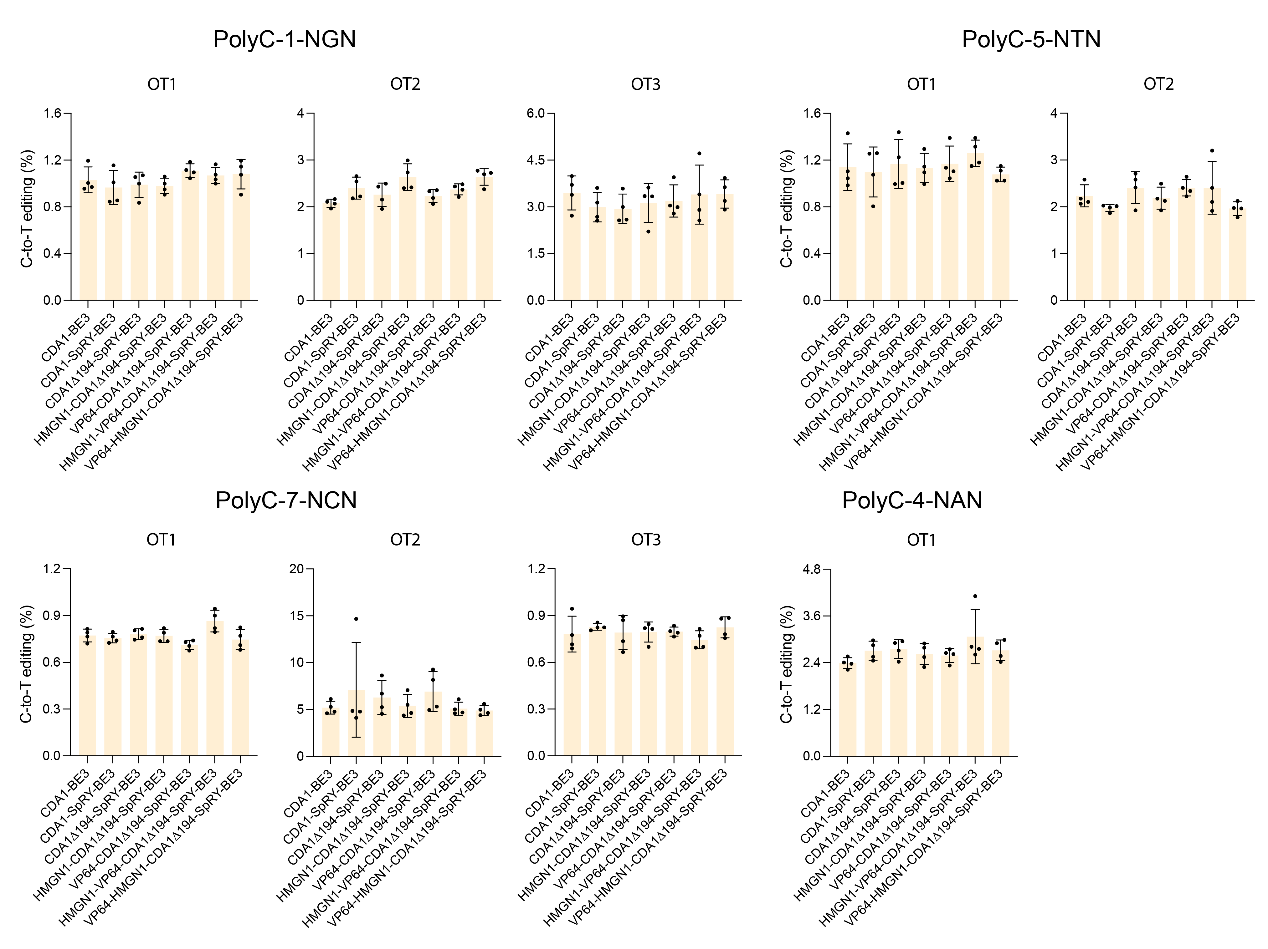


**Supplementary Figure 16**. Evaluation of sgRNA-dependent off-target editing frequencies across various base editor constructs. The bar charts display the C-to-T editing percentages at nine potential off-target sites predicted by Cas-OFFinder for the targets PolyC-1-NGN, PolyC-5-NTN, PolyC-7-NCN, and PolyC-4-NAN. The designations OT1, OT2, and OT3 represent distinct potential off-target sequences evaluated for each primary target locus. The x-axis of each panel sequentially presents the off-target editing frequencies mediated by CDA1-BE3, CDA1-SpRY-BE3, CDA1Δ194-SpRY-BE3, HMGN1-CDA1Δ194-SpRY-BE3, VP64-CDA1Δ194-SpRY-BE3, HMGN1-VP64-CDA1Δ194-SpRY-BE3, and VP64-HMGN1-CDA1Δ194-SpRY-BE3. The individual black dots represent data from three independent biological replicates (n=3), and the error bars indicate the mean ± standard deviation. Source data are provided as a Source Data file.


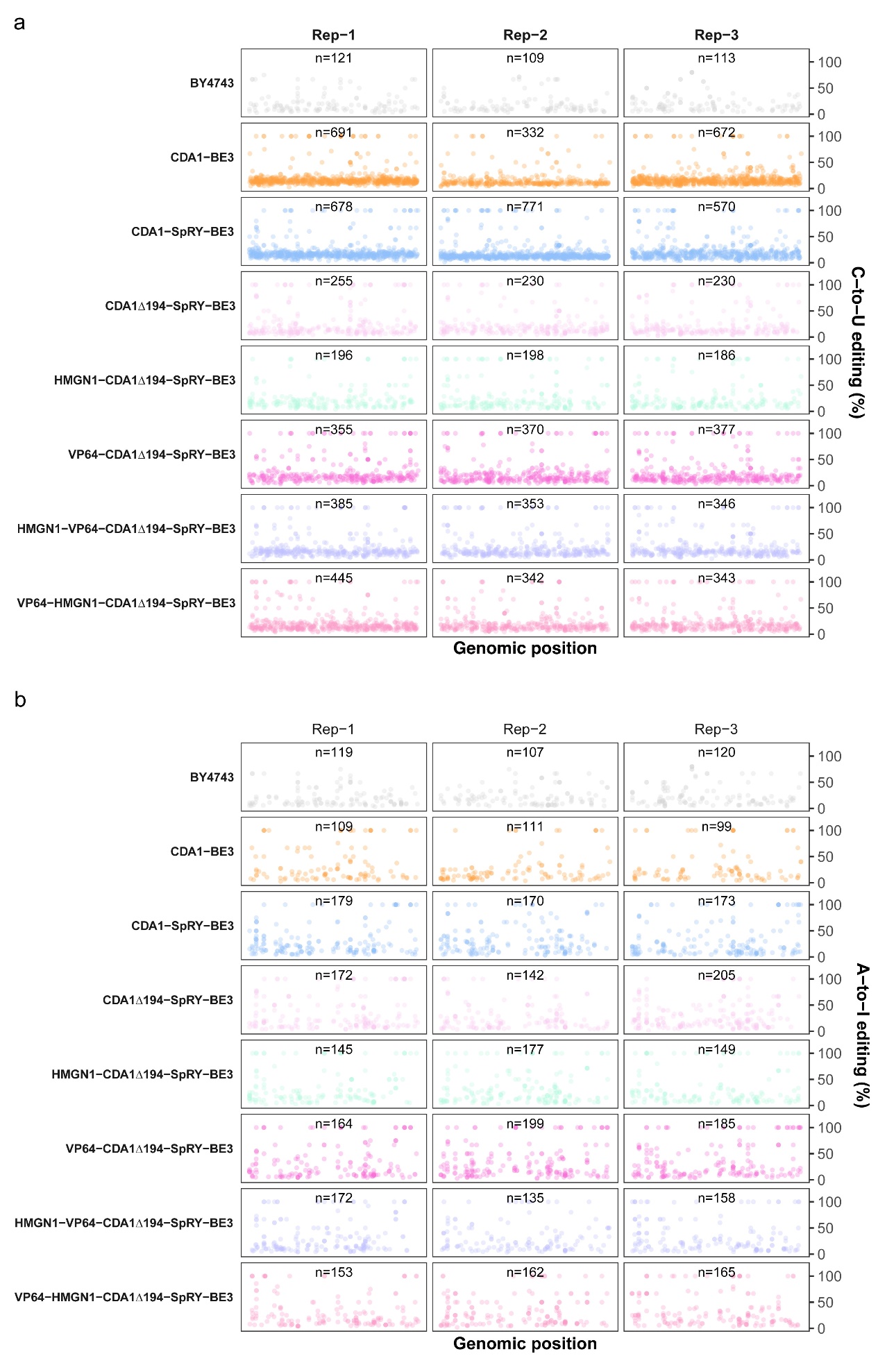


**Supplementary Figure 17.** Transcriptome-wide evaluation of RNA off-target editing induced by various base editors. Transcriptome sequencing was performed to assess genetic changes in the BY4743 strain expressing CDA1-BE3, CDA1-SpRY-BE3, CDA1Δ194-SpRY-BE3, HMGN1-CDA1Δ194-SpRY-BE3, VP64-CDA1Δ194-SpRY-BE3, HMGN1-VP64-CDA1Δ194-SpRY-BE3, and VP64-HMGN1-CDA1Δ194-SpRY-BE3, alongside the wild-type BY4743 strain serving as a negative control lacking a base editor construct. **a** Scatter plots displaying the transcriptome-wide C-to-U RNA editing efficiencies. In each panel, the x-axis indicates the genomic position, and the y-axis indicates the C-to-U editing efficiency percentage. **b** Scatter plots displaying the transcriptome-wide A-to-I RNA editing efficiencies. The x-axis indicates the genomic position, and the y-axis indicates the A-to-I editing efficiency percentage. For all panels, each dot represents an identified off-target event, and the value n indicates the total number of these off-target events. The three biological replicates are arranged horizontally for each tested group.
